# Structural basis of SUFU–GLI interaction in human Hedgehog signalling regulation

**DOI:** 10.1107/S0907444913028473

**Published:** 2013-11-19

**Authors:** Amy L. Cherry, Csaba Finta, Mikael Karlström, Qianren Jin, Thomas Schwend, Juan Astorga-Wells, Roman A. Zubarev, Mark Del Campo, Angela R. Criswell, Daniele de Sanctis, Luca Jovine, Rune Toftgård

**Affiliations:** a Department of Biosciences and Nutrition and Center for Biosciences, Karolinska Institutet, Novum, Hälsovägen 7, SE-141 83 Huddinge, Sweden; b Department of Medical Biochemistry and Biophysics, Karolinska Institutet, Scheeles väg 2, SE-171 77 Stockholm, Sweden; c Biomotif AB, Enhagsvägen 7, SE-182 12 Danderyd, Sweden; d Rigaku Americas Corporation, 9009 New Trails Drive, The Woodlands, TX 77381, USA; e Structural Biology Group, European Synchrotron Radiation Facility, 6 Rue Jules Horowitz, 38043 Grenoble, France

**Keywords:** SUFU, GLI, Hedgehog signalling regulation

## Abstract

Hedgehog signalling plays a fundamental role in the control of metazoan development, cell proliferation and differentiation, as highlighted by the fact that its deregulation is associated with the development of many human tumours. SUFU is an essential intracellular negative regulator of mammalian Hedgehog signalling and acts by binding and modulating the activity of GLI transcription factors. Despite its central importance, little is known about SUFU regulation and the nature of SUFU–GLI interaction. Here, the crystal and small-angle X-ray scattering structures of full-length human SUFU and its complex with the key SYGHL motif conserved in all GLIs are reported. It is demonstrated that GLI binding is associated with major conformational changes in SUFU, including an intrinsically disordered loop that is also crucial for pathway activation. These findings reveal the structure of the SUFU–GLI interface and suggest a mechanism for an essential regulatory step in Hedgehog signalling, offering possibilities for the development of novel pathway modulators and therapeutics.

## Abstract 2

Crystal and small-angle X-ray scattering structures of full-length human SUFU alone and in complex with the conserved SYGHL motif from GLI transcription factors show major conformational changes associated with binding and reveal an intrinsically disordered region crucial for pathway activation.

## 1. Introduction   

The Hedgehog (Hh^1^) signalling pathway plays a key role in directing cellular growth and tissue patterning during embryonic development. Furthermore, in normal adult physiology the pathway is implicated in stem cell maintenance, tissue repair and regeneration. Perturbations in the pathway thus lead to a wide range of developmental deficiencies and have been implicated in several types of human cancers (Nieuwenhuis & Hui, 2005 ▶; Jiang & Hui, 2008 ▶; Varjosalo & Taipale, 2008 ▶; Barakat *et al.*, 2010 ▶; Teglund & Toftgård, 2010 ▶; Ingham *et al.*, 2011 ▶).

The start and end of the signalling cascade have been well defined and are essentially conserved across species. A Hh ligand binds to the membrane receptor Patched (Ptc), which in the unliganded state represses another transmembrane protein, Smoothened (Smo). This repression is relieved upon ligand binding, allowing active Smo to regulate transcription-factor activity. In mammals, it is the family of glioblastoma (Gli) zinc-finger transcription factors, Gli1, Gli2 and Gli3, that execute pathway activation and repression at the transcriptional level. The intermediate steps of the pathway are less well understood and diverge significantly between species. The major mammalian regulator of Gli activity is the tumour suppressor protein Suppressor of fused (Sufu; Kogerman *et al.*, 1999 ▶; Dunaeva *et al.*, 2003 ▶; Merchant *et al.*, 2004 ▶; Cheng & Yue, 2008 ▶). Whilst being completely dispensable for *Drosophila* embryogenesis, this protein is absolutely essential for mammalian development, since knockout of *Sufu* in mice leads to continuous ligand-independent Hh signalling activity and embryonic lethality at ∼E9.5 (Cooper *et al.*, 2005 ▶; Svärd *et al.*, 2006 ▶). Furthermore, loss of human SUFU activity is associated with multiple cancer forms. Germline *SUFU* mutations have been found in patients with medulloblastoma (Taylor *et al.*, 2002 ▶; Slade *et al.*, 2011 ▶; Brugières *et al.*, 2012 ▶), meningioma (Aavikko *et al.*, 2012 ▶; Kijima *et al.*, 2012 ▶) and associated with Gorlin syndrome (Pastorino *et al.*, 2009 ▶; Kijima *et al.*, 2012 ▶), a condition that creates a predisposition to basal cell carcinoma. Moreover, somatic mutations and loss have been found in medulloblastoma, chondrosarcoma and rhabdomyosarcoma (Taylor *et al.*, 2002 ▶; Tostar *et al.*, 2006 ▶; Tarpey *et al.*, 2013 ▶).

Suggested models of how Sufu regulates Gli include its sequestration in the cytoplasm (Kogerman *et al.*, 1999 ▶), the recruitment of a co-repressor complex to Gli-responsive promoter regions of DNA (Cheng & Bishop, 2002 ▶) and promotion of the conversion from activator to repressor forms of Gli2 and Gli3 (Wang *et al.*, 2010 ▶). Direct binding of Sufu to all three Gli transcription factors has been well documented (Kogerman *et al.*, 1999 ▶; Pearse *et al.*, 1999 ▶; Stone *et al.*, 1999 ▶; Dunaeva *et al.*, 2003 ▶; Merchant *et al.*, 2004 ▶) and dissociation of the Sufu–Gli complex is a proposed key step in pathway activation (Humke *et al.*, 2010 ▶; Tukachinsky *et al.*, 2010 ▶). The exact nature of this interaction has however not been elucidated, although Gli-binding properties have been ascribed to both the N- and C-terminal regions of Sufu. In order to define the molecular details of the Sufu–Gli interaction and advance our knowledge of its regulation, we initiated a structural analysis of these key components at the very basis of human HH signalling.

## 2. Methods   

### 2.1. Protein expression and purification   

Bacterial expression constructs were assembled by subcloning different SUFU_32–483_ variants into vector pLJMBP4c (Monné *et al.*, 2008 ▶). MBP-SUFU-Δ and MBP-SUFU-SH plasmids were generated by replacing amino acids 279–360 with the heptamer sequence PSRGEDP and a shuffled IDR sequence, respectively (Supplementary Table S1*a* 2). The mutant MBP-SUFU_R386A,R388A,H391A,R393A_ was obtained through the GeneCust Europe DNA mutagenesis service. MBP-SUFU constructs were expressed in *Escherichia coli* JM109 (DE3) (Promega) at 21°C. Protein expression was induced at a cell density of OD_550_ = 0.5–1 with 0.1 m*M* IPTG for 16–18 h. Cells from 1 l culture were suspended in 10 ml 50 m*M* Tris–­HCl pH 7.5, 50 m*M* NaCl, 1 m*M* MgCl_2_, 1 m*M* DTT, 0.2 mg ml^−1^ lysozyme, 25 U ml^−1^ Benzonase (Sigma–Aldrich) and cOmplete Mini EDTA-free protease inhibitors (Roche) and were disrupted using three freeze–thaw cycles. Bacterial debris was removed by centrifugation at 18 000*g* for 30 min. Cleared lysates were loaded onto 5 ml HisTrap HP columns (GE Healthcare). Following extensive washing with 50 m*M* Tris–HCl pH 7.5, 1 *M* NaCl, 20 m*M* imidazole, 1 m*M* DTT, bound proteins were eluted with 50 m*M* Tris–HCl pH 7.5, 50 m*M* NaCl, 500 m*M* imidazole, 1 m*M* DTT. The eluate was concentrated to 2–3 ml using Amicon Ultra centrifugal filter units (Millipore) and further purified by size-exclusion chromatography (SEC) on a HiPrep 26/60 Sephacryl S-200 HR gel-filtration column (GE Healthcare) equilibrated with 10 m*M* Tris–HCl pH 7.4, 50 m*M* NaCl, 1 m*M* DTT. Pooled fractions containing monomeric MBP-SUFU protein were concentrated as above and filtered using 0.2 µm Ultrafree-MC centrifugal filter units (Millipore). Concentrated proteins were flash-frozen in liquid N_2_ and stored at −80°C.

Insect-cell expression of N-terminally His_6_-tagged SUFU_30–484_ was performed using the Bac-to-Bac Baculovirus Expression System (Invitrogen). Generation of recombinant baculovirus stock was carried out according to the supplier’s recommendations. Sf9 cells (Invitrogen) were cultured at 27°C in SF-900 II Serum Free Medium (Invitrogen) and infected with recombinant baculovirus at mid-logarithmic phase (10^6^ cells ml^−1^, viability ≥99%) using a multiplicity of infection of 1. After 72 h, cells were harvested by centrifugation and stored at −80°C until use. Pelleted cells from 1 l culture were lysed in 20 ml 50 m*M* Tris–HCl pH 7.4, 150 m*M* NaCl, 5 m*M* MgCl_2_, 0.2% NP-40, 1 m*M* DTT, 25 U ml^−1^ Benzonase and cOmplete Mini EDTA-free protease inhibitors (Roche) using three freeze–thaw cycles. Following centrifugation at 20 000*g* for 1 h, cleared lysate was loaded onto a 5 ml HisTrap HP column (GE Healthcare). After extensive washing with 50 m*M* Tris–HCl pH 7.5, 1 *M* NaCl, 20 m*M* imidazole, 1 m*M* DTT, bound proteins were eluted with 50 m*M* Tris–HCl pH 7.5, 1 *M* NaCl, 500 m*M* imidazole, 1 m*M* DTT. The eluate was loaded onto a HiPrep 26/60 Sephacryl S-200 HR column (GE Healthcare) equilibrated with 10 m*M* Tris–HCl pH 7.4, 1 *M* NaCl, 1 m*M* DTT. The protein was purified, concentrated, filtered and stored as above.

### 2.2. Crystallization   

MBP-SUFU-FL protein (12 mg ml^−1^ in 10 m*M* Tris–HCl pH 7.5, 50 m*M* NaCl, 1 m*M* DTT, 1 m*M* maltose) was crystallized at 4°C by hanging-drop vapour diffusion with 0.2 *M* potassium/sodium tartrate, 0.1 *M* bis-tris propane pH 8.5, 16%(*v*/*v*) PEG 3350 (at a protein:mother liquor ratio of 2:1). X-shaped crystals of 0.5 mm in width appeared after 5 d. Crystals were transferred stepwise to a cryosolution equivalent to the mother liquor plus an additional 4%(*v*/*v*) PEG 3350 and 10%(*v*/*v*) MPD, mounted in cryoloops and flash-cooled in liquid N_2_.

Crystal form I of MBP-SUFU-Δ (6.5 mg ml^−1^ in 10 m*M* Tris–HCl pH 7.5, 50 m*M* NaCl, 1 m*M* DTT, 1 m*M* maltose) was obtained at 4°C by hanging-drop vapour diffusion with 0.08 *M* sodium cacodylate pH 6.6, 20%(*v*/*v*) glycerol, 160 m*M* calcium acetate, 9%(*v*/*v*) PEG 8000. Rod-shaped crystals of 200 µm in length appeared after 14 d. Specimens were washed in a solution equivalent to the mother liquor, mounted in cryoloops and flash-cooled in liquid N_2_.

Crystal form II of MBP-SUFU-Δ (11.6 mg ml^−1^ in 10 m*M* Tris–HCl pH 7.5, 50 m*M* NaCl, 1 m*M* DTT) was crystallized by hanging-drop vapour diffusion at 4°C with 0.1 *M* Na HEPES pH 7.5, 17%(*v*/*v*) PEG 3350, 0.2 *M* NaCl. Rod-shaped crystals appeared within days and were transferred stepwise to a cryo-solution equivalent to the growth conditions plus an additional 3%(*v*/*v*) PEG 3350 and 10%(*v*/*v*) MPD, mounted in cryoloops and flash-cooled in liquid N_2_.

MBP_A216H_K220H_-SUFU-Δ_W61D_L62S_G63F_P453A_Δ454–456_K457A_ (10 mg ml^−1^ in 10 m*M* Tris–HCl pH 7.5, 50 m*M* NaCl, 1 m*M* DTT, 1 m*M* maltose) was mixed in a 1:1 molar ratio with zinc acetate and a 1:4 molar ratio with GLI1_p_ or GLI3_p_. Complexes were crystallized by hanging-drop vapour diffusion at 4°C with 1:1 or 2:1 drops of protein:well solution [14–18%(*v*/*v*) PEG 3350, 0.2 *M* sodium formate]. Irregular-shaped crystals which grew out of precipitate were transferred stepwise to a cryo-solution equivalent to the growth conditions plus an additional 10%(*v*/*v*) PEG 3350, mounted in cryoloops and flash-cooled in liquid N_2_.

### 2.3. X-ray data collection   

Diffraction data were collected at the European Synchrotron Radiation Facility (ESRF), Grenoble, France. Data were collected from single crystals at 100 K using the following beamlines and wavelengths: MBP-SUFU-FL, ID14-1 (Wakatsuki *et al.*, 1998 ▶), 0.9334 Å; MBP-SUFU-Δ crystal form I, ID23-1 (Nurizzo *et al.*, 2006 ▶), 1.0723 Å; MBP-SUFU-Δ crystal form II, ID23-2 (Flot *et al.*, 2010 ▶), 0.8726 Å; MBP_A216H_K220H_-SUFU-Δ_W61D_L62S_G63F_P453A_Δ454–456_K457A_–GLI1_p_ and MBP_A216H_K220H_-SUFU-Δ_W61D_L62S_G63F_P453A_Δ454–456_K457A_–GLI3_p_, ID29 (de Sanctis *et al.*, 2012 ▶), 0.9762 Å (Table 1 ▶).

### 2.4. X-ray structure determination   

The MBP-SUFU-FL and MBP-SUFU-Δ data sets were processed with *iMosflm* (Battye *et al.*, 2011 ▶) and integrated with *SCALA* (Evans, 2006 ▶) and *TRUNCATE* (French & Wilson, 1978 ▶); the MBP_A216H_K220H_-SUFU-Δ_W61D_L62S_G63F_P453A_Δ454–456_K457A_–GLI1_p_ and MBP_A216H_K220H_-SUFU-Δ_W61D_L62S_G63F_P453A_Δ454-456_K4–7A_–GLI3_p_ data sets were processed using *XDS* (Kabsch, 2010 ▶) in the *xia*2 pipeline (Winter, 2010 ▶).

The structure of MBP-SUFU-FL was solved by molecular replacement (MR) with *Phaser* (McCoy *et al.*, 2007 ▶) using an initial data set collected at 4.1 Å resolution {overall *R*_p.i.m._ = 13.4%; outer shell [4.10–4.32 Å, 〈*I*/σ(*I*)〉 = 2.1] *R*_p.i.m._ = 37.9%}; the search models were the MBP molecule from chain *A* of PDB entry 3d4g (residues Thr3–Ala371; Monné *et al.*, 2008 ▶) and the N-terminal domain of SUFU from chain *A* of PDB entry 1m1l (residues Pro32–Asp262; Merchant *et al.*, 2004 ▶). As in the case of MBP-ZP3 (Monné *et al.*, 2008 ▶), correctness of the solution was confirmed by clear difference electron density for a molecule of d-(+)-maltose (coordinates for which were not included in the search ensemble) within the ligand-binding pocket of the two MBP molecules in the asymmetric unit. The structure of the SUFU C-terminal domain was manually built into σ_A_-weighted difference Fourier maps (Read, 1986 ▶) using *Coot* (Emsley *et al.*, 2010 ▶). Refinement against a maximum-likelihood (ML) target was performed with *phenix.refine* (Adams *et al.*, 2010 ▶). Simulated annealing was initially used with a starting temperature of 5000 K, and translation/libration/screw (TLS) refinement of *B* factors was performed during the final rebuilding cycles on the basis of *TLSMD* (Painter & Merritt, 2006 ▶) analysis of individually refined atomic displacement parameters; noncrystallographic symmetry (NCS) restraints were kept during all refinement steps based on manually determined local differences between molecules. Riding H atoms were added with *phenix.reduce* and used throughout; validation was performed using *MolProbity* (Chen *et al.*, 2010 ▶). The Ramachandran statistics were 98.0% favoured, 2.0% allowed and 0.0% outliers.

The structure of MBP-SUFU-Δ crystal form I was solved by MR using the refined coordinates of MBP-SUFU-FL (MBP, Glu5–Ala371; SUFU, Pro32–Leu278, Ile361–Asp449, Glu455–Val478) as a search model. The MBP-SUFU-Δ crystal form II data were also phased by MR using the SUFU moiety of MBP-­SUFU-FL and the unliganded form of MBP (PDB entry 1omp, residues Lys1–Thr366; Sharff *et al.*, 1992 ▶) as search models; however, significant manual rebuilding was required to model three of the four MBP moieties within the asymmetric unit of this crystal (chains *B*–*D*), which are highly disordered (average *B* factor = 272 Å^2^) compared with the rest of the structure (average *B* factor = 122 Å^2^). The structure of MBP-SUFU-Δ crystal form II was refined using the refined coordinates of crystal form I of the same construct as a reference. The Ramachandran statistics for crystal form I were 98.0% favoured, 1.9% allowed and 0.1% outliers and those for crystal form II were 97.8% favoured, 1.9% allowed and 0.3% outliers.

The GLI1_p_ and GLI3_p_ complex structures were solved by MR using the refined coordinates of MBP-SUFU-Δ as a search model; as in the case of the latter the structures were refined and validated essentially as described for MBP-SUFU. The Ramachandran statistics for the GLI1_p_ complex were 97.9% favoured, 2.0% allowed and 0.1% outliers and those for the GLI3_p_ complex were 97.9% favoured, 2.1% allowed and 0.0% outliers. Figures were generated with *PyMOL* (Schrödinger) and *LigPlot* ^+^ (Laskowski & Swindells, 2011 ▶). Supplementary Video S1 was produced with *PyMOL* based on an interpolation calculated by *RigiMOL* (Schrödinger).

### 2.5. Limited proteolysis   

SUFU_32–484_ cloned in pLJMBP6c was expressed in *E. coli* and purified as described above. The purified protein was digested with trypsin (Sigma–Aldrich) in 100 m*M* ammonium bicarbonate at 23°C. The protease was inactivated by adding 10 m*M* PMSF and sample-loading buffer followed by heating at 95°C for 5 min. Protein bands were separated by SDS–PAGE and visualized by Coomassie Blue staining. Proteolytic peptide fragments were identified by N-terminal sequencing (Alphalyse A/S) as well as MALDI–TOF and MALDI–TOF/TOF mass-spectrometric analyses (Ultraflex II and Autoflex III, Bruker Daltonics).

### 2.6. Hydrogen–deuterium exchange (HDX)   

To compare amide HDX kinetics between MBP-SUFU-FL and MBP-SUFU-Δ, 4 µl of each protein (both at 11 mg ml^−1^ in 50 m*M* Na HEPES pH 7.0, 50 m*M* NaCl, 5 m*M* β-mercaptoethanol, 1.5 m*M* maltose) was mixed with 13 µl deuterated buffer with the same ionic composition as the protein sample. For MBP-SUFU-FL–Gli3_p_ interaction experiments, 45 µl MBP-SUFU-FL (11 mg ml^−1^) was mixed with 5 µl Gli3_p_ (6 m*M*; a 5.5-fold excess) and incubated for 1 h. In the latter case, exchange was initiated by mixing 4 µl MBP-SUFU-FL–Gli3_p_ with 13 µl deuterated buffer with the same ionic composition as the sample. Each reaction was stopped by adding 9 µl quenching solution (4 *M* urea, 50 m*M* TCEP, 1% TFA) and flash-freezing in liquid N_2_. Deuteration reactions were incubated for 60, 300, 600 and 1800 s for the MBP-SUFU-FL and MBP-SUFU-Δ experiments and 300, 600 and 1800 s for the MBP-SUFU-FL–Gli3_p_ interaction experiments (at 4°C, in triplicate). A 24 h incubation was used as a fully deuterated sample for back-exchange correction.

Samples were analyzed in a semi-automated HDX-MS system (Biomotif AB, Sweden) in which manually injected samples were automatically digested, cleaned and separated at 1.0°C. Deuterated samples were digested using a 1 min stop-flow protocol (Poroszyme Immobilized Pepsin Cartridge, Applied Biosystems, USA), followed by an online desalting step with a 1.0 × 10 mm C-18 precolumn (ACE HPLC Columns, UK) using 0.05% TFA at 300 µl min^−1^ for 3 min. Peptic peptides were then separated by a C18 Halo 2.1 × 100 mm (Advanced Materials Technology, USA) or a Chromolith FastGradient 50 × 2 mm for MBP-­SUFU-FL/MBP-SUFU-Δ and MBP-SUFU-FL–Gli3_p_ experiments, respectively. Peptic peptides were separated by a 8.5 min 5–­40% linear gradient of acetonitrile in 0.3% formic acid. An Orbitrap XL mass spectrometer (Thermo Scientific, USA) operated at 60 k resolution was used for the analysis.

Several LC MS/MS runs were carried out to identify MBP-SUFU peptic peptides. The *Mascot* software (Matrix Science) was used to search an MBP-SUFU sequence database. Peptides with scores higher than 20 were selected for HDX kinetic studies. In addition, each selected peptide was further validated by manually inspecting the MS/MS spectrum. The *HDExaminer* software (Sierra Analytics, USA) was used to process all HDX MS data.

### 2.7. Small-angle X-ray scattering (SAXS)   

SAXS data were collected on a Rigaku BioSAXS-1000 using Cu radiation (λ = 1.5418 Å) from a Rigaku FR-E+ SuperBright rotating-anode X-ray generator. The BioSAXS-1000 consists of an optic, a vacuum chamber and a detector. The optic is a double-bounce Rigaku Confocal MaxFlux multilayer optic specifically designed for the BioSAXS-1000 and it focuses the X-rays to a point at the detector. The vacuum chamber contains a Kratky block, a sample holder and a beamstop with an integrated PIN diode. The Kratky block collimates the X-ray beam into the shape of a line that is approximately 0.5 mm tall at the sample position; however, the beam is focused to a point at the detector. SAXS data were recorded using a DECTRIS PILATUS 100K detector and the camera length was 0.5 m. The *q* range for all SAXS data was from 0.01 to 0.68 Å^−1^.

All samples and buffers were loaded into 1.0 mm quartz capillaries, placed under vacuum and measured at 20°C. Glucose isomerase was used as a secondary standard to assess *I*(0) data. MBP-SUFU proteins used for SAXS were purified from *E. coli* as described above, except that SEC was performed in 50 m*M* Na HEPES pH 7.0, 50 m*M* NaCl, 5 m*M* β-mercaptoethanol, 1.5 m*M* maltose and only two fractions from the peak were pooled for analysis (Supplementary Fig. S1). The proteins were 99% pure as judged by Coomassie Blue staining of an SDS–PAGE gel. SEC buffer passed through the gel-filtration column immediately prior to protein purification was used as the blank. Scattering data were measured from frozen aliquots of MBP-SUFU that were thawed on ice, centrifuged at 20 000*g* for 10 min, diluted in SEC buffer and loaded into 1.0 mm diameter quartz capillaries. For experiments with GLI peptide, MBP-SUFU-Δ was mixed in a 1:10 molar ratio with GLI1_p_ in the same buffer. Three concentrations of each protein (between 1.1 and 7.3 mg ml^−1^) were measured to ensure that concentration effects did not influence data analysis. The protein concentration was determined by measuring the absorbance at 280 nm with extinction coefficients of 1.4073 for MBP-SUFU-FL and 1.5484 for MBP-SUFU-Δ. Each SAXS profile was the result of a 60 min exposure in image-refreshing mode. In this mode, a 60 min exposure was the sum of six consecutive 10 min exposures that were individually inspected to ensure that radiation damage was not present in the sample.

Initial data analysis, reduction of scattering images to one-dimensional plots of intensity *versus* momentum transfer (*q*) followed by buffer subtraction, was performed by the Rigaku *SAXSLab* software package. Buffer-subtracted data were then analyzed using the *ATSAS* program suite (Petoukhov *et al.*, 2012 ▶). Radius of gyration (*R*_g_) values were determined from Guinier plots in *PRIMUS* (Konarev *et al.*, 2003 ▶) and pair distance distribution functions, *P*(*r*), were computed with *GNOM* (Svergun, 1992 ▶). *V*_c_, *Q*_R_ and χ^2^_free_ were calculated according to Rambo & Tainer (2013 ▶). *Ab initio* molecular envelopes of MBP-SUFU were generated using *DAMMIF* (Franke & Svergun, 2009 ▶). For MBP-SUFU-FL and MBP-SUFU-Δ, 15 independent *DAMMIF* models were aligned, averaged and filtered using *DAMAVER* (Volkov & Svergun, 2003 ▶) and potential clusters were assessed with *DAMCLUST* (Petoukhov *et al.*, 2012 ▶). For MBP-SUFU-FL there were clearly two clusters, each composed of seven models; the mean normalized spatial discrepancy for the averaging of cluster 1 was 0.728 ± 0.050 and that for cluster 2 was 0.729 ± 0.063. For MBP-SUFU-Δ there was only a single cluster and the mean normalized spatial discrepancy for the averaging was 0.722 ± 0.031. *CRYSOL* (Svergun *et al.*, 1995 ▶) was used to calculate the theoretical SAXS profile from each of the MBP-SUFU crystal structure models and compare it with the experimental SAXS profiles. The C^α^-atom coordinates of SUFU residues that are disordered in the MBP-SUFU-FL crystal structure were modelled by performing nine independent runs of *CORAL* (Petoukhov & Svergun, 2005 ▶; mean χ^2^/χ^2^_free_ values of 10.3 ± 1.6 and 14.8 ± 2.4), assuming the parts of MBP-SUFU-Δ remain fixed and using data to *q* = 0.3 Å^−1^. Crystal structures and *CORAL* models were initially aligned to the averaged SAXS envelopes using *SUPCOMB* and were manually adjusted by rotation and translation thereafter. The model of MBP-SUFU-Δ that had the best fit to the average SAXS envelope was assembled by fusing crystallographic models of maltose-bound MBP and SUFU-Δ in one specific relative orientation observed in the 2.8 Å resolution apo structure. The seven-residue loop which replaces the IDR in SUFU-Δ was then grafted from one of the molecules in the 3.5 Å resolution structure and energy-minimized with *YASARA Structure *(Krieger *et al.*, 2002 ▶) so that it properly fitted the gap. The resulting optimized model agrees with the scattering data as well as the original crystallographic models of MBP-SUFU-Δ do.

### 2.8. Microscale thermophoresis   

A 5-FAM-labelled peptide (FAM-GLI1_p_) comprising residues 115–131 of GLI1 (Supplementary Table S1*b*) was synthesized by Pepceuticals Ltd. A titration series of each recombinant protein was prepared by serial 1:1 dilutions in MST buffer (50 m*M* Tris–HCl pH 7.6, 150 m*M* NaCl, 10 m*M* MgCl_2_, 0.05% Tween-20) and mixed in a 1:1 ratio with FAM-GLI1_p_ to give final concentrations of 50 n*M* FAM-GLI1_p_ with MBP-SUFU constructs in the ranges 14.2–29 070 and 54–110 500 n*M*. Reactions were aspirated into glass capillaries and the thermophoretic movement of FAM-GLI1_p_ was monitored using a Monolith NT.115 instrument (NanoTemper Technologies; Wienken *et al.*, 2010 ▶), with a laser on for 30 s and off for 5 s and a laser power/voltage of 20% for MBP-SUFU-FL and the MBP-SUFU-FL mutants, 40% for MBP-SUFU-Δ and 80% for MBP-SUFU-SH. Fluorescence was measured before laser heating (*F*_Cold_) and after 30 s of laser-on time (*F*_Hot_). The normalized fluorescence *F*_Hot_/*F*_Cold_ reflects the concentration ratio of labelled molecules. *F*_Hot_/*F*_Cold_ was plotted directly and multiplied by a factor of 10, yielding a relative change in fluorescence per thousand. *K*_d_ was calculated from three independent thermophoresis measurements using *NanoTemper* software (NanoTemper Technologies).

### 2.9. Production of Shh conditioned medium   

293 EcR Shh cells (ATCC; Cooper *et al.*, 1998 ▶) were cultured in DMEM high-glucose (4.5 g l^−1^) l-glutamine medium (PAA Laboratories GmbH) supplemented with 10% foetal bovine serum (FBS; Saveen Werner), 0.1 m*M* MEM non-essential amino acids (Sigma–Aldrich), 1 m*M* sodium pyruvate (Sigma–Aldrich), 100 units ml^−1^ penicillin and 100 µg ml^−1^ streptomycin (PAA Laboratories GmbH). At 90–100% confluency, cells were switched to medium containing 2% FBS and Shh production was induced with 1.5 µ*M* ponasterone A (Enzo Life Sciences). Conditioned medium was collected after 24 h of induction, filtered through 0.22 µm filters, flash-frozen in liquid N_2_ and stored at −80°C.

### 2.10. Thermal stability assays   

Recombinant MBP-SUFU proteins were diluted to 2.6 µ*M* in 50 m*M* Na HEPES pH 7.0, 50 m*M* NaCl, 5 m*M* β-mercaptoethanol; the peptides GLI1_p_ and GLI1_p_-SH (Supplementary Table S1*b*), synthesized by Dr W. Mawby (University of Bristol), were dissolved in the same buffer. Proteins were spun at 14 000*g* for 20 min at 4°C before adding SYPRO Orange (Molecular Probes) to a final concentration of 6×. Reaction volumes of 25 µl were prepared in 96-well PCR plates with 2.2 µ*M* protein/SYPRO Orange solution and, where applicable, 44 µ*M* peptide. Plates were sealed with optical tape, heated from 20 to 90°C in 12 s 0.2°C steps in an iCycler and fluorescence was detected using an excitation wavelength of 470 nm and an emission wavelength of 570 nm.

### 2.11. Fluorescence experiments   

Recombinant MBP-SUFU constructs were diluted to 2.5 µ*M* in 50 m*M* Na HEPES pH 7.0, 50 m*M* NaCl, 5 m*M* β-­mercaptoethanol and spun at 14 000*g* for 20 min at 4°C before the addition of SYPRO Orange to a final 5× concentration of dye. The fluorescence of 100 µl samples was measured in black 96-well plates (Nunc) using excitation at λ = 470 nm and emission at λ = 570 nm. GLI1_p_ or GLI1_p_-SH peptides prepared in the same buffer as above were added in the given ratios and mixed before a second reading was taken. Readings post-peptide addition were divided by the corresponding pre-peptide addition measurements to obtain normalized results.

### 2.12. Co-immunoprecipitation and immunoblot analysis   

Cos-7 cells were transfected separately with C-terminally FLAG-tagged GLI1 in pCMV5 (Andersson *et al.*, 1989 ▶) and N-terminally Myc-tagged SUFU in pCMV-Script (Stratagene) using Fugene 6 (Roche). 24 h after transfection, the cells were lysed with 50 m*M* Tris–HCl pH 7.4, 150 m*M* NaCl, 1% NP-40, 0.25% sodium deoxycholate, 1 m*M* *N*-ethylmaleimide (Fluka), 1 m*M* dithiothreitol and cOmplete Mini protease inhibitors (Roche). Normalized lysates were combined and pre-incubated for 6 h. Co-immunoprecipitation was performed for 16 h using agarose-conjugated anti-FLAG M2 (Sigma–Aldrich) and anti-Myc 9b11 (Cell Signaling) antibodies. The beads were washed three times with 500 µl lysis buffer and the bound protein was eluted by boiling the beads in SDS–PAGE sample buffer and analyzed by Western blotting. Samples were separated by SDS–PAGE, transferred to polyvinylidene fluoride Immobilon-P membranes (Millipore) and probed using anti-Myc 71D10 (Cell Signaling) or anti-Myc 9E10 (Santa Cruz) antibodies and anti-DYKDDDDK tag antibody (Cell Signaling). Similar results were obtained in four independent experiments.

### 2.13. Hh pathway activity measurements   

SUFU variants with mutated IDR regions, the SUFU-Δ, SUFU-SH, SUFU-SH2 and SUFU-IDR_fly_ constructs, were generated by replacing amino acids 279–360 of N-terminally Myc-tagged full-length SUFU in pcDNA3.1 (Invitrogen) with corresponding sequences synthesized at GenScript USA Inc. (Supplementary Table S1*a*). HEK 293 cells were transfected with a mixture of GLI1-FLAG or Myc-GLI2 (Roessler *et al.*, 2005 ▶; a kind gift from Erich Roessler) and Myc-SUFU constructs together with 12GLI-RE-TKO-luc luciferase reporter (Kogerman *et al.*, 1999 ▶) and pRL-SV40 (Promega) internal control using Fugene 6 or X-tremeGENE 9 (Roche) transfection reagent. Expression assays were carried out for 48 h, followed by luciferase activity measurements using a Dual Luciferase Activity Assay Kit (Promega). To verify the expression levels of transfected SUFU and GLI constructs, aliquots of cell lysates were separated by SDS–PAGE and subjected to immunoblot analysis as above. The densities of the visualized protein bands were quantified using the *ImageJ* 1.47v image-analysis and image-processing software (Schneider *et al.*, 2012 ▶).

*Sufu* ^−/−^ mouse embryonic fibroblast (MEF) cells (Svärd *et al.*, 2006 ▶) were transfected with a mixture of p8x3′Gli-BS LucII or p8x3′Gli-mBS LucII reporter constructs (Sasaki *et al.*, 1997 ▶; generous gifts from Hiroshi Sasaki) together with pRL-SV40 (Promega) internal control using Lipofectamine LTX with *Plus* reagent (Invitrogen) according to the manufacturer’s instructions. Following transfection, *Sufu* ^−/−^ MEF cells were grown until the culture reached confluency (24–48 h) and the cells were then switched to low-serum medium (0.5% FBS) with 100–200 n*M* SAG (kindly provided by Jan Bergman, Karolinska Institutet), 10 µ*M* purmorphamine (Calbiochem) or Shh conditioned medium (1:4) and grown for an additional 48–72 h. Cell lysis and luciferase activity measurements were performed using a Dual Luciferase Activity Assay Kit (Promega). For statistical evaluation of reporter gene assays, a one-tailed paired Student’s t-test was used. All activity measurements were performed at least three times in independent experiments.

## 3. Results   

### 3.1. Structure of human SUFU   

**Figure 1:**
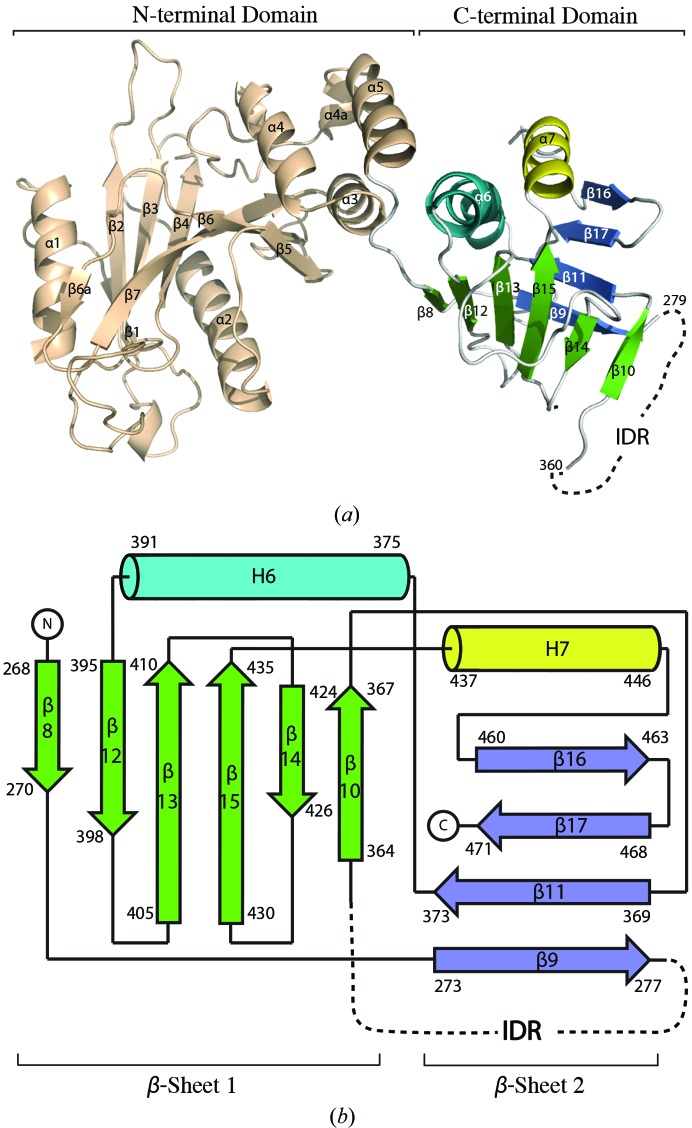
Overall structure and topology of full-length human SUFU. (*a*) Crystal structure of SUFU with the N-terminal domain coloured beige and the C-­terminal domain coloured according to (*b*). (*b*) Topology scheme, with the intrinsically disordered region (IDR) represented by a dashed line.

**Figure 2:**
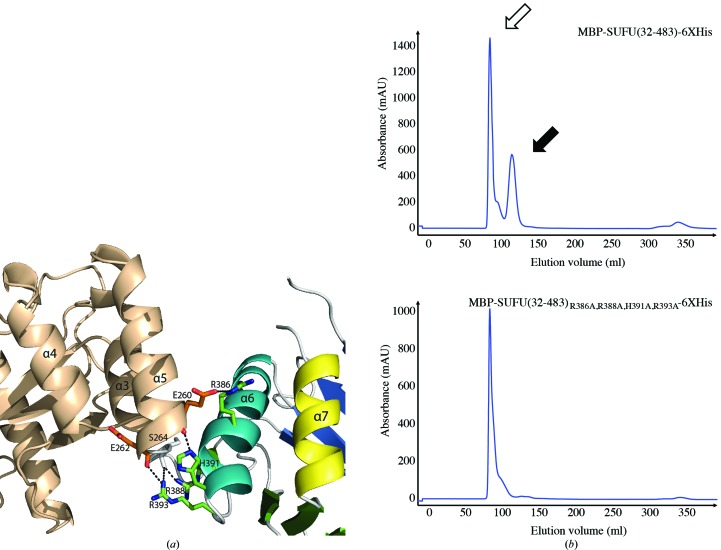
Interaction between the N- and C-terminal domains is essential for the solubility of heterologous SUFU. (*a*) Interactions between residues in the five-helix bundle formed between the two domains. (*b*) Size-exclusion chromatography profiles; the open and filled arrows indicate peaks corresponding to aggregated protein eluted with the void volume and soluble monomeric protein, respectively.

Although a crystal structure of an N-terminal domain of SUFU has been reported (Merchant *et al.*, 2004 ▶), no information is available on its C-terminal region and how this is arranged relative to the rest of the protein owing to difficulties in producing soluble full-length recombinant protein. Here, we have expressed an essentially full-length human SUFU construct in *E. coli* by fusing it to an N-terminal maltose-binding protein (MBP) molecule *via* a three-alanine linker (Smyth *et al.*, 2003 ▶; Monné *et al.*, 2008 ▶). The fusion (MBP-SUFU-FL), containing residues 32–483 of human SUFU and a C-terminal hexahistidine tag, was purified by immobilized metal ion affinity chromatography and size-exclusion chromatography (SEC), crystallized in the presence of maltose and the structure was determined to 3.0 Å resolution (*R* = 20.0%, *R*_free_ = 24.6%; Fig. 1 ▶ *a* and Table 2 ▶). The N-terminal domain exhibits the same structure as previously determined (Merchant *et al.*, 2004 ▶), despite the presence of the N-terminal MBP fusion partner. The C-terminal region folds into a domain that comprises a four-stranded and a six-stranded β-­sheet, both with mixed topologies, which are connected by two antiparallel α-helices (Figs. 1 ▶ *a* and 1 ▶ *b*). This is a novel protein fold, with only weak structural similarity (*DALI* *Z*-­scores of 2.7–2.8; Holm & Rosenström, 2010 ▶) to proteosomal Jab1/MPN domain proteins (PDB entries 1oi0 and 1r5x; Tran *et al.*, 2003 ▶; Ambroggio *et al.*, 2004 ▶). The first helix in the C-terminal domain of SUFU (helix 6) is bent and interacts with helix 5 *via* residues Arg386, Arg388, His391 and Arg393 (Fig. 2 ▶ *a*), thus forming a five-helix bundle comprising three helices from the N-terminal domain and two helices from the C-terminal domain. These interactions explain why the C-­terminal domain could not be expressed alone and are consistent with the observation that mutation of the above residues causes protein aggregation, probably owing to misfolding (Fig. 2 ▶ *b*).

### 3.2. SUFU contains an intrinsically disordered region   

Although the crystallized protein was intact (Supplementary Fig. S2*a*), both SUFU molecules in the orthorhombic asymmetric unit show no apparent density for the C-terminal domain residues 279–360, suggesting that this region of the molecule is highly mobile. Since disorder often impacts crystal diffraction quality, we generated a new construct (MBP-SUFU-Δ) in which these 82 residues were replaced with a shorter seven-residue loop (Supplementary Table S1*a*). This construct was expressed and purified in the same way as MBP-SUFU-FL and crystallized both in the presence (crystal form I) and absence (crystal form II) of maltose. The corresponding structures were determined to 2.8 Å resolution (*R* = 20.0%, *R*_free_ = 23.4%) and 3.5 Å resolution (*R* = 25.9%, *R*_free_ = 29.3%), respectively (Table 2 ▶). Both crystal forms contain four molecules in the asymmetric unit, with pairs assuming a head-to-tail arrangement identical to that observed in the structure of MBP-SUFU-FL (Supplementary Fig. S2*b*). The fold of MBP-SUFU-Δ is essentially the same as for the full-length protein, demonstrating that the disordered residues are not critical for protein folding or overall stability.

**Figure 3:**
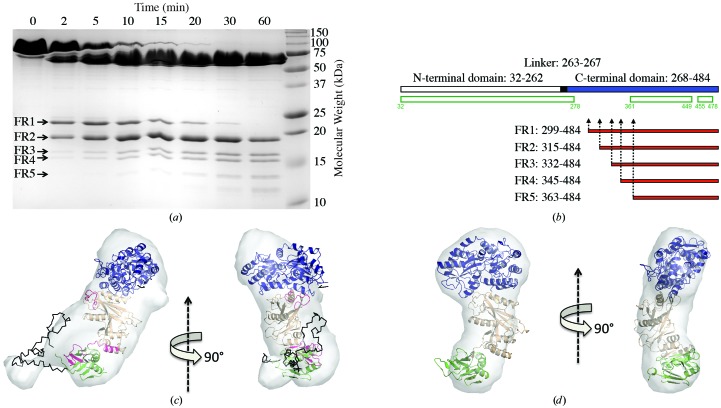
SUFU contains an intrinsically disordered region. (*a*, *b*) Trypsin digest analysis of MBP-SUFU-FL. Open green rectangles indicate structured parts of the protein in our crystallographic model. The red rectangles FR1–FR5 represent proteolytic fragments. (*c*, *d*) Fit of MBP-SUFU-FL and MBP-SUFU-Δ crystal structures into average *ab initio* envelopes calculated from SAXS data. MBP, blue; SUFU N-terminal domain, beige; SUFU C-terminal domain, green. Residues of the IDR, built by *CORAL*, are shown in black. Peptides that are more protected from HDX in MBP-SUFU-FL than in MBP-SUFU-Δ are shown in pink.

To determine whether the unobserved region in the C-­terminal domain is also disordered in solution, we subjected MBP-SUFU-FL to limited proteolysis with trypsin (Receveur-Bréchot *et al.*, 2006 ▶). Several protease-hypersensitive sites were found clustered within residues 299–363, a stretch almost exactly overlapping the region of missing density in the crystal structure (Figs. 3 ▶ *a* and 3 ▶ *b*). Likewise, hydrogen/deuterium-exchange (HDX) analysis (Brock, 2012 ▶; Brudler *et al.*, 2006 ▶) of the same region shows that it is highly sensitive to deuteration (Supplementary Figs. S3*a* and 4). Moreover, limited proteolytic fragment patterns of human SUFU expressed in insect and bacterial cells are essentially identical (Supplementary Fig. S5). Collectively, these data suggest that residues 279–360 constitute an intrinsically disordered region (IDR) that is an inherent feature of native human SUFU. In cases such as these when crystallography only reveals part of the picture, SAXS is a useful complementary technique which can be used to examine the conformation of disordered regions in solution (Putnam *et al.*, 2007 ▶). Interestingly, SAXS comparison of MBP-SUFU-FL and MBP-SUFU-Δ indicates that the IDR forms a flexible protrusion from the C-terminal domain that covers its β-sheet 1 (Figs. 3 ▶ *c* and 3 ▶ *d*, Supplementary Fig. S6 and Supplementary Table S2). The latter is confirmed by the HDX data, which show protection of the same sheet as well as an N-terminal domain loop exposed on the same surface of the molecule in MBP-SUFU-FL but not in MBP-SUFU-Δ (Fig. 3 ▶ *c* and Supplementary Figs. S3 and 4).

**Figure 4:**
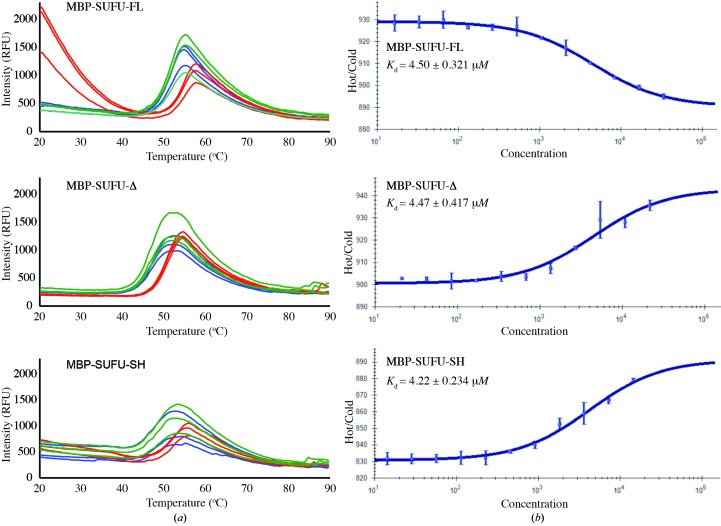
The SUFU IDR has distinct structural properties. (*a*) Thermal stability assays of MBP-SUFU constructs performed in triplicate, either alone (blue) or with GLI1_p_ (red) or GLI1_p_-SH (green). All constructs bind to GLI1_p_; however, MBP-SUFU-FL has different physical properties upon initial GLI1_p_ binding, as shown by the marked increase in fluorescence. (*b*) Microscale thermophoresis experiments with FAM-GLI1_p_ and titrated MBP-SUFU constructs, showing an average of three separate experiments. All proteins have similar affinity, but the thermophoretic properties of FAM-GLI1_p_ are modified differently between the MBP-SUFU-FL construct and the MBP-SUFU-Δ and MBP-SUFU-SH constructs, reflecting a difference in shape.

To determine whether the IDR affects the GLI-binding properties of SUFU, we used a human GLI1-derived peptide (GLI1_p_; residues 115–131; Supplementary Table S1*b*) containing the highly conserved SYGH motif important for interaction with SUFU (Dunaeva *et al.*, 2003 ▶) in a thermal stability assay with MBP-SUFU-FL and MBP-SUFU-Δ (Fig. 4 ▶ *a*). The addition of GLI1_p_ stabilized both proteins: *T*_m_ for MBP-SUFU-FL was shifted by 4°C and that for MBP-SUFU-Δ was shifted by 3.6°C. No stabilization was provided by a control peptide comprising the same residues randomly shuffled (GLI1_p_-SH; Supplementary Table S1*b*). Interestingly, MBP-SUFU-FL exhibited high initial fluorescence values upon addition of GLI1_p_, but no such effect was observed with either MBP-SUFU-Δ or a third construct which was identical to MBP-SUFU-FL except that the 82 residues of the IDR were shuffled (MBP-SUFU-SH; Supplementary Table S1*a*). The GLI1 dose dependency of this effect was confirmed in a separate experiment with increasing GLI1_p_ concentrations (Supplementary Fig. S7*a*). Furthermore, MBP-SUFU-FL was more stable (*T*_m_ = 50.1°C) than both the MBP-SUFU-Δ (*T*_m_ = 46.6°C) and MBP-SUFU-SH (*T*_m_ = 47.7°C) constructs. Taken together, these data show that despite being apparently disordered the IDR has properties which are different from those of a random loop and alter upon GLI1_p_ peptide binding.

The affinity of the three SUFU constructs for GLI1_p_ was determined more accurately using a FAM-labelled GLI1_p_ peptide (FAM-GLI1_p_; Supplementary Table S1*b*) in microscale thermophoresis (Wienken *et al.*, 2010 ▶) experiments (Fig. 4 ▶ *b*). The *K*_d_ values derived for all three constructs were comparable; however, the thermophoretic properties of the peptide were modified differently: whereas binding to MBP-SUFU-FL increased the rate of movement in the thermophoretic gradient, binding to MBP-SUFU-Δ or MBP-SUFU-SH decreased it. Since thermophoretic mobility is affected by the molecular charge, size and solvation shell (Wienken *et al.*, 2010 ▶) and because MBP-SUFU-FL and MBP-SUFU-SH have the same theoretical size and charge, these data further suggest that the native IDR behaves differently to a random sequence of residues upon GLI1_p_ binding.

Additional evidence of the distinct structural properties of the IDR was provided by SDS–PAGE analysis of SUFU expressed in either mammalian cells or bacteria. Despite having identical amino-acid composition, full-length SUFU (SUFU-FL) displays different electrophoretic mobility to two SUFU constructs in which IDR residues are alternatively shuffled (SUFU-SH and SUFU-SH2; Supplementary Table S1*a* and Supplementary Figs. S7*b* and 7*c*). Anomalous mobility in SDS–PAGE has been described for proteins with post-translational modifications, atypical amino-acid composition and disordered segments (Iakoucheva *et al.*, 2001 ▶). Mass-spectrometric analysis has not revealed any post-translational modifications in MBP-SUFU-FL peptides, including peptides involving Ser342 and Ser346 (data not shown), which are residues that have been reported to be targets for phosphoryl­ation in mammalian cells (Chen *et al.*, 2011 ▶). Together, these data suggest that disparities in electrophoretic migrations are most likely to be owing to distinct structural properties of the native IDR.

### 3.3. Structure of SUFU in complex with GLI peptides   

**Figure 5:**
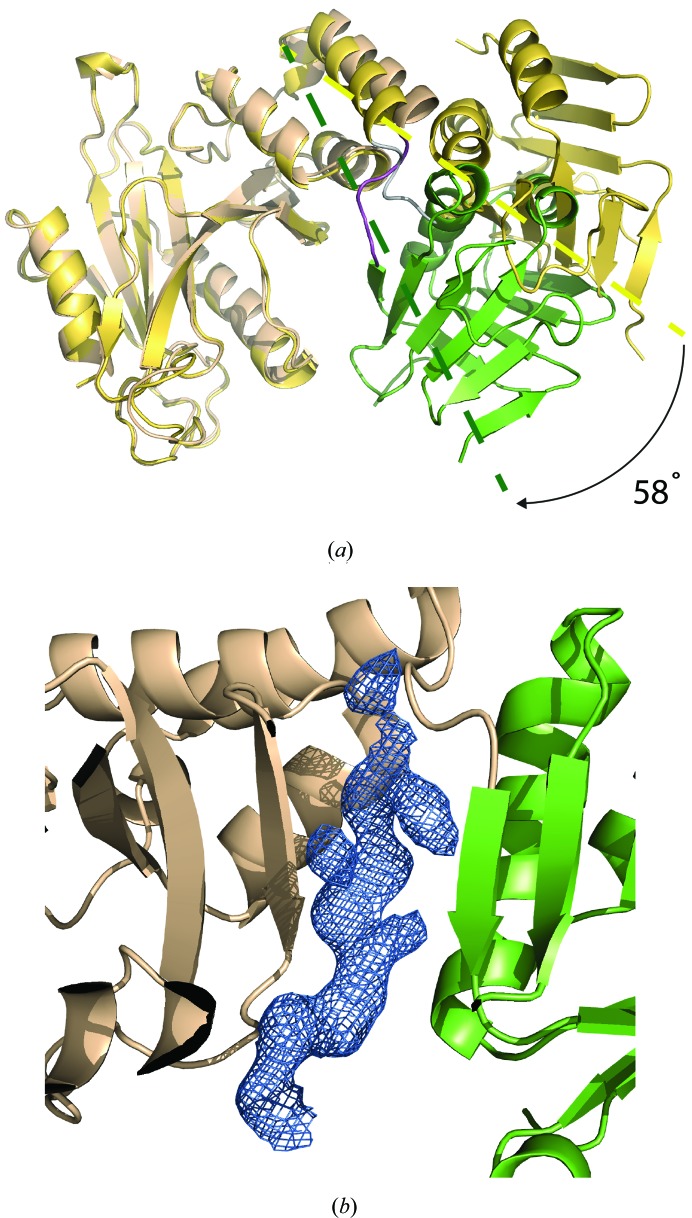
Crystal structure of MBP_A216H_K220H_-SUFU-Δ_W61D_L62S_G63F_P453A_Δ454–456_K457A_ in complex with GLI3_p._ (*a*) Superposition of the N-terminal domains of apo (yellow with grey linker) and peptide-bound (beige, N-­terminal domain; green, C-terminal domain; purple linker) structures shows a 58° rotation of the C-terminal domain *via* a flexible linker. (*b*) The position of GLI3_p_ in the MBP-_216H_220H__-SUFU-Δ_W61D_L62S_G63F_P453A_Δ454–456_K457A_–peptide co-crystal. An averaged kick OMIT map contoured at 3.0σ shows well defined density for GLI3_p_ lying between the β-­sheets of the SUFU N-terminal domain (beige) and C-terminal domain (green).

**Figure 6:**
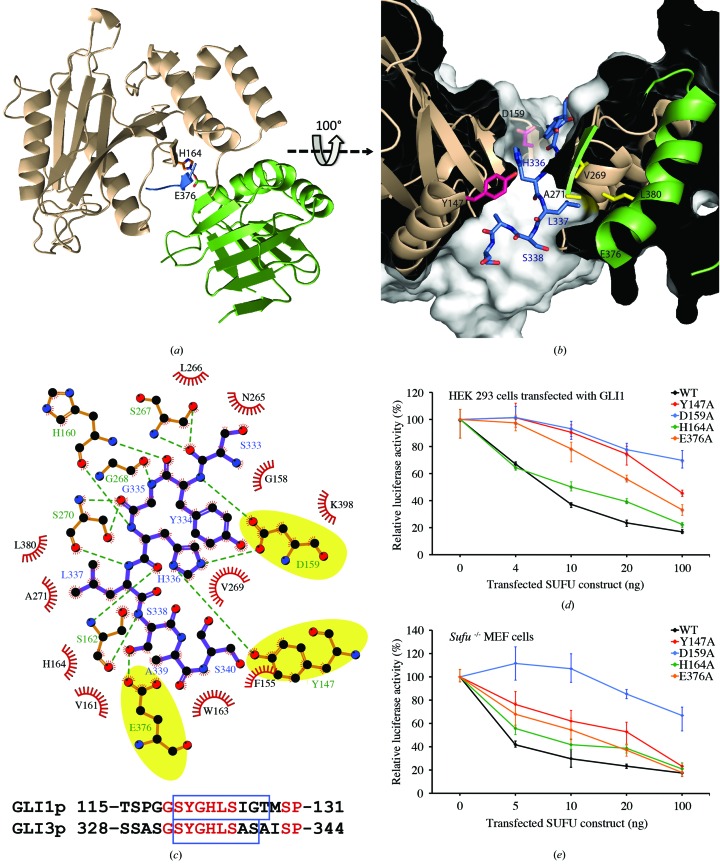
Mechanism of GLI binding. (*a*, *b*, *c*) Interactions between MBP_A216H_K220H_-SUFU-Δ_W61D_L62S_G63F_P453A_Δ454–456_K457A_ and GLI3_p_. (*a*) The peptide (blue) is clamped between the N-terminal (beige) and C-terminal (green) domains. (*b*) GLI3_p_ (blue residue labels) binds in a channel with His336 and Lys337 protruding into deep pockets. (*c*) SUFU–GLI3_p_ interactions, with side-chain hydrogen bonds highlighted in yellow, and comparison of GLI1_p_ and GLI3_p_. Residues that are conserved in GLI1, GLI2 and GLI3 are shown in red. Blue boxes indicate residues with visible electron density. (*d*, *e*) Mutations around the GLI3_p_ binding site have varying effects on the ability of SUFU-FL to repress GLI1-induced reporter gene activity in HEK 293 cells (*d*) and to suppress constitutive pathway activity in *Sufu* ^−/−^ cells (*e*).

**Figure 7:**
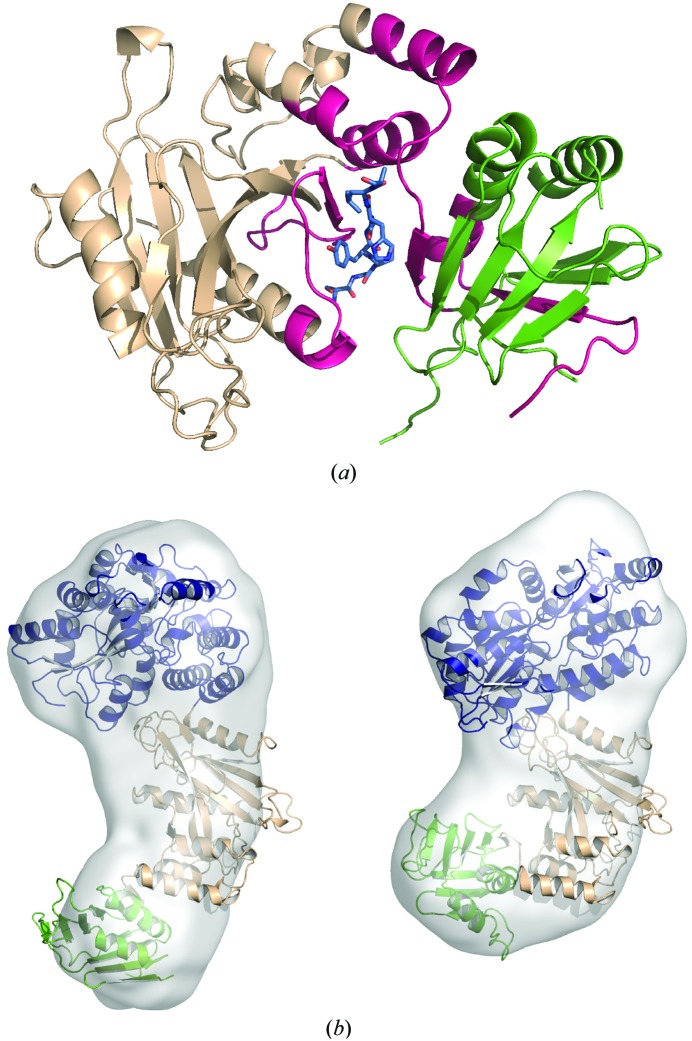
MBP-SUFU and GLI peptides interact in the same way in solution as in crystals. (*a*) HDX analysis of MBP-SUFU-FL with GLI3_p_ (blue). Areas that are more protected from exchange in the presence of the peptide are highlighted in pink. (*b*) Comparison of SAXS envelopes for MBP-SUFU-Δ in the absence (left) and presence (right) of GLI1_p_. Crystal structures of apo MBP-SUFU-Δ and of MBP_A216H_K220H_-SUFU-Δ_W61D_L62S_G63F_P453A_Δ454–456_K457A_ co-crystallized with GLI3_p_ superpose well on apo and holo SAXS envelopes, respectively.

In order to determine how SUFU interacts with GLI transcription factors, we attempted to co-crystallize MBP-SUFU-FL and MBP-SUFU-Δ with GLI1_p_ as well as corresponding peptides from human GLI2 (GLI2_p_; residues 267–283) and GLI3 (GLI3_p_; residues 328–344) (Supplementary Table S1*b*). Despite extensive screening, these attempts were unsuccessful. Therefore, the residues WLG61–63 of SUFU were mutated to DSF in MBP-SUFU-Δ to disrupt crystal contacts between a loop within the SUFU N-terminal domain and residues in the C-terminal domain (Supplementary Fig. S2*b*) previously implicated in GLI binding (Merchant *et al.*, 2004 ▶). Furthermore, another flexible loop in SUFU was shortened and residues 216 and 220 in the MBP moiety were mutated to histidine in order to promote metal ion-mediated crystallization of the fusion protein (Laganowsky *et al.*, 2011 ▶). The resulting construct, MBP_A216H_K220H_-SUFU-Δ_W61D_L62S_G63F_P453A_Δ454–456_K457A_, produced crystals with GLI1_p_ and GLI3_p_ that diffracted to 2.8 Å resolution (*R* = 19.7%, *R*_free_ = 23.4% and *R* = 20.1%, *R*_free_ = 23.4%, respectively). Crystals with both peptides belonged to space group *P*2_1_ and contained four molecules in the asymmetric unit which all exhibited a rotation, *via* a flexible linker, of 58° relative to the apo crystal structures (Fig. 5 ▶ *a* and Supplementary Video S1). Each molecule had clear density for the peptide between domains (Fig. 5 ▶ *b*). Peptide modelled into this density forms a β-strand clamped between the two domains, creating one continuous 13-strand β-sheet spanning both domains. Interactions between SUFU His164 and Glu376 secure the closed conformation (Fig. 6 ▶ *a*). HDX protection analysis and SAXS experiments confirmed that this protein/peptide conformation occurs in solution and is not a crystallization artifact (Fig. 7 ▶, Supplementary Table S2 and Supplementary Figs. S6, S8 and S9). In both structures the GLI peptide fits snugly into a narrow channel with the histidine from the SYGH motif (Dunaeva *et al.*, 2003 ▶; His123 in GLI1 and His336 in GLI3) protruding into a pocket where it forms hydrogen-bonding interactions with Tyr147 and Asp159 (Figs. 6 ▶ *b* and 6 ▶ *c* and Supplementary Table S3). The mutation of Tyr147, Asp159 or Glu376 in MBP-SUFU-FL abolished detectable binding to GLI1_p_ in the microscale thermophoresis assay (data not shown). To determine whether these binding differences were translated into functional differences in the cell, we examined the transcriptional activity of GLI1 in HEK 293 cells transiently transfected with mutated SUFU constructs (Fig. 6 ▶ *d*). The mutation of Asp159 and Tyr147 had a significant effect on the ability of SUFU to repress GLI, whereas the mutation of Glu376 and His164 had a smaller effect. A similar pattern was observed in experiments measuring constitutive Hh pathway activity in *Sufu* ^−/−^ MEFs (Fig. 6 ▶ *e*). Notably, the leucine immediately following the GLI SYGH motif, which is also completely conserved, packs tightly into a hydrophobic pocket formed by SUFU residues Val269, Ala271 and Leu380 (Fig. 6 ▶ *b*). The following serine (conserved except in *Xenopus* and *Ciona*) is hydrogen bonded to Glu376. In agreement with these observations, a GLI3 peptide that terminates at His336 (GLI3_p_-SHC; residues 328–336; Supplementary Table S1*b*) is unable to protect MBP-SUFU-FL from deuteration in HDX experiments (data not shown). Hence, the critical binding motif extends beyond that previously described (Dunaeva *et al.*, 2003 ▶).

### 3.4. Regulatory role of the SUFU IDR   

**Figure 8:**
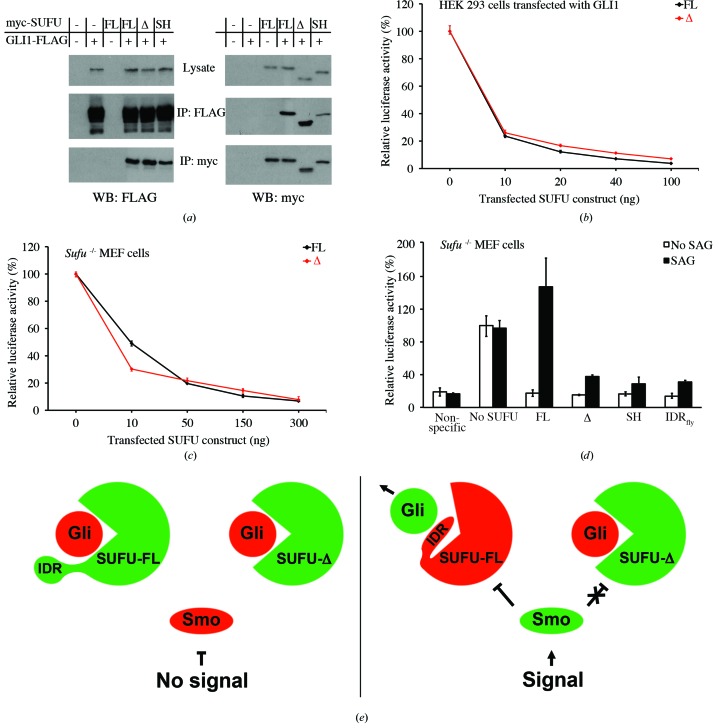
The IDR regulates SUFU activity. (*a*) SUFU-FL (FL), SUFU-Δ (Δ) and SUFU-SH (SH) all bind GLI1 when co-expressed in Cos-7 cells, as shown by Co-IP. (*b*, *c*) SUFU-FL and SUFU-Δ repress GLI1-induced reporter gene activity in HEK 293 cells (*b*) and suppress constitutive pathway activity in *Sufu* ^−/−^ cells (*c*) with similar efficiency. (*d*) SUFU-Δ, SUFU-SH and SUFU-IDR_fly_ (IDR_fly_) expressed in *Sufu* ^−/−^ cells are unable to mediate Hh pathway reactivation induced by the SMO agonist SAG. Error bars indicate the range of data in three parallel samples. (*e*) Schematic model of the regulatory role of SUFU with and without an intact IDR. Green and red colours indicate activated and repressed states of proteins, respectively.

The observed physical differences between MBP-SUFU constructs with and without the IDR suggest a functional role of this domain. In agreement with the thermophoresis data, there was no remarkable difference in GLI1 binding observed in co-immunoprecipitation (Co-IP) experiments with SUFU-FL, SUFU-SH and SUFU-Δ (Fig. 8 ▶ *a*). Similarly, transcriptional activity induced by both GLI1 and GLI2 was efficiently repressed by SUFU-FL, SUFU-Δ and SUFU-SH in transient transfection assay experiments (Fig. 8 ▶ *b* and Supplementary Figs. S10*a* and S10*b*), and deletion of the IDR had no considerable effect on repression of the constitutive Hh pathway activity in *Sufu* ^−/−^ MEFs (Fig. 8 ▶ *c*). Collectively, these results imply that the IDR in SUFU is dispensable for GLI binding and repression activity in cells without upstream pathway activation.

The versatile nature of IDRs makes them ideal for the formation of protein–protein interactions, and intrinsically disordered stretches often function as regulatory platforms in proteins (Babu *et al.*, 2011 ▶). Hence, we set out to test whether the SUFU IDR has a role in pathway reactivation in *Sufu* ^−/−^ MEFs. While activation of cells with SMO agonist (SAG), a Hh pathway activator upstream to SUFU, overrode repression by the full-length protein, it failed to reactivate the pathway in the presence of SUFU-Δ or SUFU-SH (*p* ≤ 0.005, *n* = 10 and *p* ≤ 0.01, *n* = 4, respectively; Fig. 8 ▶ *d*). Purmorphamine, another small-molecule SMO agonist, as well as Sonic Hedgehog (Shh) had the same disparate effects on SUFU-FL and SUFU-Δ repressive function (*p* ≤ 0.05, *n* = 5 and *p* ≤ 0.005, *n* = 9 for purmorphamine and Shh, respectively; Supplementary Figs. S10*c* and S10*d*). Collectively, these results are in agreement with a simple model in which Hh activation in mammalian cells is achieved *via* an IDR-dependent repression of SUFU function (Fig. 8 ▶ *e*).

The role of Sufu in the regulation of Hh signalling has diverged between vertebrates and invertebrates (Varjosalo *et al.*, 2006 ▶). Whereas mammalian SUFU is a major negative regulator and knockout of its gene is lethal (Cooper *et al.*, 2005 ▶; Svärd *et al.*, 2006 ▶), *Drosophila* Sufu has only a minor role and loss-of-function mutations have no phenotype (Préat, 1992 ▶). Sequence alignment reveals that the IDR is the most divergent region between human and *Drosophila* Sufu, with only 11% sequence conservation compared with 42% in the rest of the protein. However, despite the lack of sequence conservation, this region of *Drosophila* Sufu is also predicted to have relatively little secondary structure (Supplementary Fig. S11). To test whether the regulatory role of SUFU IDR is conserved between human and fly, we created a chimeric SUFU construct in which amino acids 279–360 of human SUFU were replaced with the corresponding region from *Drosophila* Sufu (residues 275–340; SUFU-IDR_fly_; Supplementary Table S1*a*). Similarly to SUFU-Δ and SUFU-SH, SUFU-IDR_fly_ was able to repress GLI activity, but repression could not be relieved by the addition of SAG (Fig. 8 ▶ *d*). Hence, the evolution of the IDR in SUFU may be closely linked to the differing role of Sufu between species.

## 4. Discussion   

In this study, we have determined the structure of full-length human SUFU, an essential negative regulator of mammalian Hedgehog signalling, alone and in complex with GLI peptides representing the major conserved SUFU interaction partners. The data provide new mechanistic insights into the inner workings of one of the key signalling pathways governing tissue patterning during embryonic development and determining cell fate and phenotype. Hitherto available knowledge at the structural and biophysical level has mainly been focused on receptor components in the upper part of the pathway (Beachy *et al.*, 2010 ▶), including the recent description of the structure of the GPCR-like receptor protein SMO (Wang *et al.*, 2013 ▶). In contrast, little is known about the evolutionarily conserved intracellular core pathway components that act further downstream, such as SUFU and GLI. A structure of the N-terminal half of SUFU was reported (Merchant *et al.*, 2004 ▶) that is confirmed in the present study, whereas for GLI only the structure of the DNA-binding zinc-finger domain is known (Pavletich & Pabo, 1993 ▶).

Studies aimed at the identification of protein regions involved in Sufu–Gli interaction have suggested that the N- and C-terminal regions of Sufu interact separately with the C- and N-terminal regions of Gli, respectively (Ding *et al.*, 1999 ▶; Merchant *et al.*, 2004 ▶; Barnfield *et al.*, 2005 ▶). In contrast, the structures of our SUFU–GLI peptide complexes show that both the N- and C-terminal halves of SUFU interact simultaneously with a major evolutionarily conserved SUFU-binding motif, including the amino acids SYGH, within the N-­terminal half of GLI (Dunaeva *et al.*, 2003 ▶). Importantly, recent studies have established the regulated dissociation of Sufu and Gli2/3 as a central step in the triggering of pathway activity by Hh ligands in a manner dependent on the presence of intact primary cilia (Humke *et al.*, 2010 ▶; Tukachinsky *et al.*, 2010 ▶). Our present results reveal an intriguing mechanism at the molecular level for the regulation of this fundamental step in the mammalian Hh signalling pathway (Fig. 8 ▶ *e*). We show that in the unbound form SUFU adopts an open conformation in which the IDR hovers over the surface of SUFU that interacts with the N-terminal domain of GLI proteins. Upon GLI binding, SUFU undergoes a large conformational change in which the N- and C-terminal domains come together to clamp highly conserved GLI residues in the middle of a large β-sheet (Supplementary Video S1). The important functional role of amino acid Asp159 in the N-terminal domain of SUFU, as shown here and in the study by Merchant *et al.* (2004 ▶), is explained by critical hydrogen-bonding interactions with a conserved histidine residue in GLI (Figs. 6 ▶ *b* and 6 ▶ *c*). Moreover, the tight packing of the leucine next to the histidine in GLI with SUFU residues, coupled with the importance of this leucine in the protection of SUFU from deuteration, strongly suggest that the minimal SUFU binding motif in GLI encompasses the amino acids SYGHL.

Of particular interest is the finding that SUFU contains an intrinsically disordered domain that is rearranged upon GLI peptide binding. This suggests that SUFU acts as a central signal organizer in a protein-interaction network in which the IDR plays a key role in modulation of allostery or regulated autoinhibition, properties that are found to be common among proteins with intrinsic disorder and that hence exist in many different structural states (Ferreon *et al.*, 2013 ▶; Trudeau *et al.*, 2013 ▶). The observed regulatory role of the SUFU IDR in relaying an HH signal may thus be owing to an allosteric function induced by post-translational modifications or interaction of the IDR with a new partner protein causing a change in the binding affinity between SUFU and GLI. Alternatively, it is possible that the IDR has a role in determining intra­cellular localization or that the SUFU IDR may act as an inhibitory module with regard to SUFU–GLI binding, as suggested by the observation that in the structure of SUFU alone the IDR appears to shield the GLI-binding surface of the protein. We propose that the IDR acts as a gatekeeper, which in the rearranged conformation becomes a target for HH-dependent regulatory factors facilitating the release of GLI from SUFU.

A remaining challenge is to identify the signals involved and to understand at the molecular level how activation of SMO couples to SUFU–GLI dissociation. Interestingly, amino-acid residues present within the IDR can serve as targets for phosphorylation by PKA and GSK3β (Ser342 and Ser346) and for ubiquitylation (Lys321) (Chen *et al.*, 2011 ▶; Kim *et al.*, 2011 ▶). However, the phosphorylation of these residues does not appear to be induced by the HH ligand and rather leads to a stabilization of SUFU (Chen *et al.*, 2011 ▶), implying a possible role in determining the level of overall HH responsiveness. The functional implication of the ubiquitination of Lys321 is presently unknown.

In evolutionary terms, it appears that whereas the Sufu–Gli association promoting generation of Gli repressor forms is conserved, the relative importance and regulatory mechanisms have diverged (Ingham *et al.*, 2011 ▶). In line with this view, we find that fly sufu is nonfunctional in mammalian cells (data not shown) and in particular that the predicted fly IDR is unable to functionally replace its human counterpart. Consistently, fly smo is unable to mediate transcriptional Hh pathway activation in mammalian cells in culture (Bijlsma *et al.*, 2012 ▶). These observations suggest that divergence of the regulatory mechanism impinging on the Sufu IDR is a major factor underlying the species difference in Sufu function.

SUFU is a tumour suppressor protein that is found to be inactivated most frequently (up to 50%) by germline mutations in children below the age of three presenting with medulloblastoma of the desmoplastic/nodular subtype (Slade *et al.*, 2011 ▶; Brugières *et al.*, 2012 ▶), whereas a somatic mutation frequency of about 10% has been reported for sporadic medulloblastomas of the desmoplastic subtype (Taylor *et al.*, 2002 ▶). Moreover, germline or somatic mutations in the *SUFU* gene have recently been described in association with meningioma and chondrosarcoma (Aavikko *et al.*, 2012 ▶; Kijima *et al.*, 2012 ▶; Tarpey *et al.*, 2013 ▶), again involving tissue types in which HH signalling is known to play a central role in normal development. As expected for a tumour suppressor, the vast majority of mutations are truncating and only two missense mutations have so far been reported. In a family predisposed to meningiomas, an R123C mutation segregated with tumour development and loss of the wild-type allele was detected in all tumours analyzed (Aavikko *et al.*, 2012 ▶). This mutation eliminates hydrogen bonding to Asp182 and Gln199, suggesting a negative effect on SUFU folding and/or stability that is consistent with the reduced inhibitory activity observed in cellular assays. M141R, a second germline missense mutation detected in a young child with a medulloblastoma of the extensive nodularity subtype (Brugières *et al.*, 2012 ▶), affects hydrophobic interactions between an α-helix and a β-sheet within the N-terminal half of SUFU. This also suggests an indirect effect of the amino-acid substitution on SUFU–GLI interaction.

Drug development efforts aimed at inhibiting the HH pathway in tumour cells have hitherto been focused on the GPCR-like receptor SMO. Unfortunately, the clinical effect of drugs that target this protein may only last for a few months (Rudin *et al.*, 2009 ▶) owing to the rapid insurgence of drug-resistant cancer cells carrying mutations in SMO itself (Yauch *et al.*, 2009 ▶). Moreover, a number of mechanisms, in addition to SUFU mutations, that induce HH pathway activation independently of the ligand/receptor level or the presence of primary cilia have been described. This suggests that components at the bottom of the pathway may constitute a better target for the treatment of cancers dependent on active HH signalling. The detailed structural description of the SUFU–GLI complex and the identification of the SUFU IDR as a key regulatory module reported here open precisely this possibility. At the same time, they provide information that could be exploited to develop novel approaches for transient activation of the pathway in the regenerative medicine setting.

## Supplementary Material

PDB reference: MBP-SUFU-FL, 4bl8

PDB reference: MBP-SUFU-Δ, crystal form I, 4bl9

PDB reference: crystal form II, 4bla

PDB reference: MBP_A216H_K220H_-SUFU-Δ_W61D_L62S_G63F_P453A_Δ454–456_K457A_–GLI1_p_, 4blb

PDB reference: MBP_A216H_K220H_-SUFU-Δ_W61D_L62S_G63F_P453A_Δ454–456_K457A_–GLI3_p_, 4bld

Supplementary Material.. DOI: 10.1107/S0907444913028473/dw5072sup1.pdf

Supplementary Video S1. Conformational change of SUFU upon GLI peptide binding. Molecular morphing between the X-ray structures of apo and GLI peptide-bound SUFU highlights the significant domain rearrangement involved in ligand binding. Protein elements are coloured as in Fig. 6(a).. DOI: 10.1107/S0907444913028473/dw5072sup2.mov

## Figures and Tables

**Table 1:** Crystallographic data-collection statistics Values in parentheses are for the highest resolution shell.

	MBP-SUFU-FL (PDB entry 4bl8)	MBP-SUFU- crystal form I (PDB entry 4bl9)	MBP-SUFU- crystal form II (PDB entry 4bla)	MBP_A216H_K220H_-SUFU-_W61D_L62S_G63F_P453A_454456_K457A_GLI1_p_ (PDB entry 4blb)	MBP_A216H_K220H_-SUFU-_W61D_L62S_G63F_P453A_454456_K457A_GLI3_p_ (PDB entry 4bld)
Beamline	ESRF ID14-1	ESRF ID23-1	ESRF ID23-2	ESRF ID29	ESRF ID29
Wavelength ()	0.9334	1.0723	0.8726	0.9762	0.9762
Temperature (K)	100	100	100	100	100
Detector	ADSC	MX-225 CCD	MX-225 CCD	PILATUS 6M-F	PILATUS 6M-F
Crystal-to-detector distance (mm)	322	380	374	568	501
Rotation range per image ()	1	1	1	0.05	0.1
Total rotation range ()	91	506	180	180	180
Exposure time per image (s)	10	0.1	2	0.04	0.04
Space group	*P*2_1_2_1_2_1_ [No. 19]	*P*1 [No. 1]	*P*2_1_2_1_2 [No. 18]	*P*2_1_ [No. 4]	*P*2_1_ [No. 4]
Unit-cell parameters					
*a* ()	97.32	93.43	117.92	116.30	116.61
*b* ()	99.55	103.28	372.57	137.60	136.55
*c* ()	192.94	111.51	86.25	116.54	116.74
()	90	63.67	90	90	90
()	90	81.13	90	105.49	105.25
()	90	76.03	90	90	90
Mosaicity ()	0.72	0.35	0.40	0.24	0.14
Resolution ()	49.783.04 (3.203.04)	30.002.80 (2.952.80)	39.583.50 (3.693.50)	62.732.80 (2.872.80)	46.362.80 (2.872.80)
Total No. of reflections	400319	676872	572793	296995	295196
No. of unique reflections	36846	87954	49033	85233	85722
Completeness (%)	99.9 (100.0)	98.6 (98.1)	99.9 (100.0)	98.2 (98.4)	99.0 (95.7)
Multiplicity	10.9 (11.1)	7.7 (7.8)	11.7 (12.5)	3.5 (3.6)	3.4 (3.4)
*I*/(*I*)	17.0 (2.0)	12.9 (2.0)	28.1 (2.2)	10.4 (1.4)†	9.6 (1.6)‡
CC_1/2_	0.999 (0.612)	0.997 (0.637)	0.999 (0.689)	0.998 (0.526)	0.996 (0.639)
*R* (%)	12.1 (139.5)	13.2 (113.6)	24.2 (202.1)	6.1 (77.1)	7.1 (66.7)
*R*_r.i.m._ (%)	12.7 (146.3)	14.1 (121.7)	25.5 (210.8)	8.2 (103.8)	9.3 (91.9)
*R*_p.i.m._ (%)	3.9 (43.7)	5.1 (43.4)	7.4 (59.5)	5.8 (71.6)	6.8 (62.2)
Overall *B* factor from Wilson plot (^2^)	102.0	78.3	91.6	94.1	89.6

† The mean *I*/(*I*) in the outer shell is 2.0 at 2.96 resolution.

‡ The mean *I*/(*I*) in the outer shell is 2.0 at 2.92 resolution.

**Table 2:** Crystallographic refinement statistics Values in parentheses are for the highest resolution shell.

	MBP-SUFU-FL (PDB entry 4bl8)	MBP-SUFU- crystal form I (PDB entry 4bl9)	MBP-SUFU- crystal form II (PDB entry 4bla)	MBP_A216H_K220H_-SUFU-_W61D_L62S_G63F_P453A_454456_K457A_GLI1_p_ (PDB entry 4blb)	MBP_A216H_K220H_-SUFU-_W61D_L62S_G63F_P453A_454456_K457A_GLI3_p_ (PDB entry 4bld)
Resolution ()	48.663.04 (3.113.04)	29.482.80 (2.862.80)	39.583.50 (3.683.50)	19.982.80 (2.872.80)	19.942.80 (2.872.80)
Completeness (%)	99.9	98.6	99.9	98.2	99.0
No. of reflections in working set	34582 (2139)	85749 (5328)	46271 (6432)	82980 (5887)	83347 (5599)
No. of reflections in test set	2198 (123)	2191 (137)	2462 (362)	1963 (151)	1975 (135)
Final *R*_cryst_ (%)	20.0 (34.8)	20.0 (33.0)	25.9 (39.3)	19.7 (29.8)	20.1 (30.0)
Final *R*_free_ (%)	24.6 (40.6)	23.4 (38.3)	29.3 (42.7)	23.4 (34.7)	23.4 (35.0)
No. of fusion protein molecules in asymmetric unit	2	4	4	4	4
No. of non-H atoms†					
Protein	11449 [5760]	22691 [11369]	22889 [11577]	22792 [11441]	22792 [11441]
Peptide	0	0	0	253	228
Maltose	46	92	0	92	92
Zn^2+^ ion	0	0	0	4	4
Total	11495	22783	22889	23141	23116
R.m.s. deviations‡					
Bond lengths ()	0.01 [0.01]	0.01 [0.01]	0.01 [0.01]	0.01 [0.01]	0.01 [0.01]
Bond angles ()	0.90 [0.97]	0.85 [0.92]	1.25 [1.25]	0.10 [1.05]	1.14 [1.11]
Chirality (^3^)	0.055 [0.062]	0.046 [0.051]	0.058 [0.059]	0.041 [0.044]	0.069 [0.067]
Planarity ()	0.004 [0.005]	0.005 [0.005]	0.011 [0.011]	0.005 [0.005]	0.006 [0.005]
Dihedral angles ()	13.39 [14.22]	12.52 [13.17]	11.90 [12.81]	11.38 [11.81]	12.48 [12.55]
*B* factors† (^2^)					
Protein	106 [109, 103]	71 [76, 67]	178 [242, 115]	100 [92, 107]	97 [91, 103]
Peptide	N/A	N/A	N/A	95	90
Maltose	73	60	N/A	67	66
Zn^2+^ ion	N/A	N/A	N/A	88	68
ML estimate for coordinate error ()/phase error ()	0.43/26.4	0.36/26.4	0.61/32.2	0.41/28.4	0.39/28.6
Ramachandran plot‡ (%)					
Favoured	98.0 [99.3, 96.8]	98.0 [98.8, 97.2]	97.8 [99.0, 96.7]	97.8 [98.8, 96.9]	97.8 [98.3, 97.4]
Allowed	2.0 [0.7, 3.2]	1.9 [1.2, 2.5]	1.9 [1.0, 2.7]	2.1 [1.2, 2.8]	2.2 [1.7, 2.6]
Outliers	0.0 [0.0, 0.0]	0.1 [0.0, 0.3]	0.3 [0.0, 0.6]	0.1 [0.0, 0.3]	0.0 [0.0, 0.0]

† Values relative to the MBP and SUFU moieties of the MBP fusion proteins are shown in square brackets.

‡ Values relative to the MBP and SUFU (including GLI peptides, if present) moieties of the MBP fusion proteins are shown in square brackets.

## Footnotes

1 Pathway components are abbreviated as follows: human, uppercase; mouse and other vertebrates in general, uppercase first letter; *Drosophila*, lowercase.

2 Supplementary material has been deposited in the IUCr electronic archive (Reference: DW5072).
