# Supplementary material for: Structural basis of SUFU–GLI interaction in human Hedgehog signalling regulation
Source: Acta Crystallogr D Biol Crystallogr. 2013 Nov 19;69(Pt 12):2563–79. doi: 10.1107/S0907444913028473 (PMC3852661; doi:10.1107/S0907444913028473)
Supplement: Supplementary file 1 [file d-69-02563-sup1.pdf]

Supplementary Material

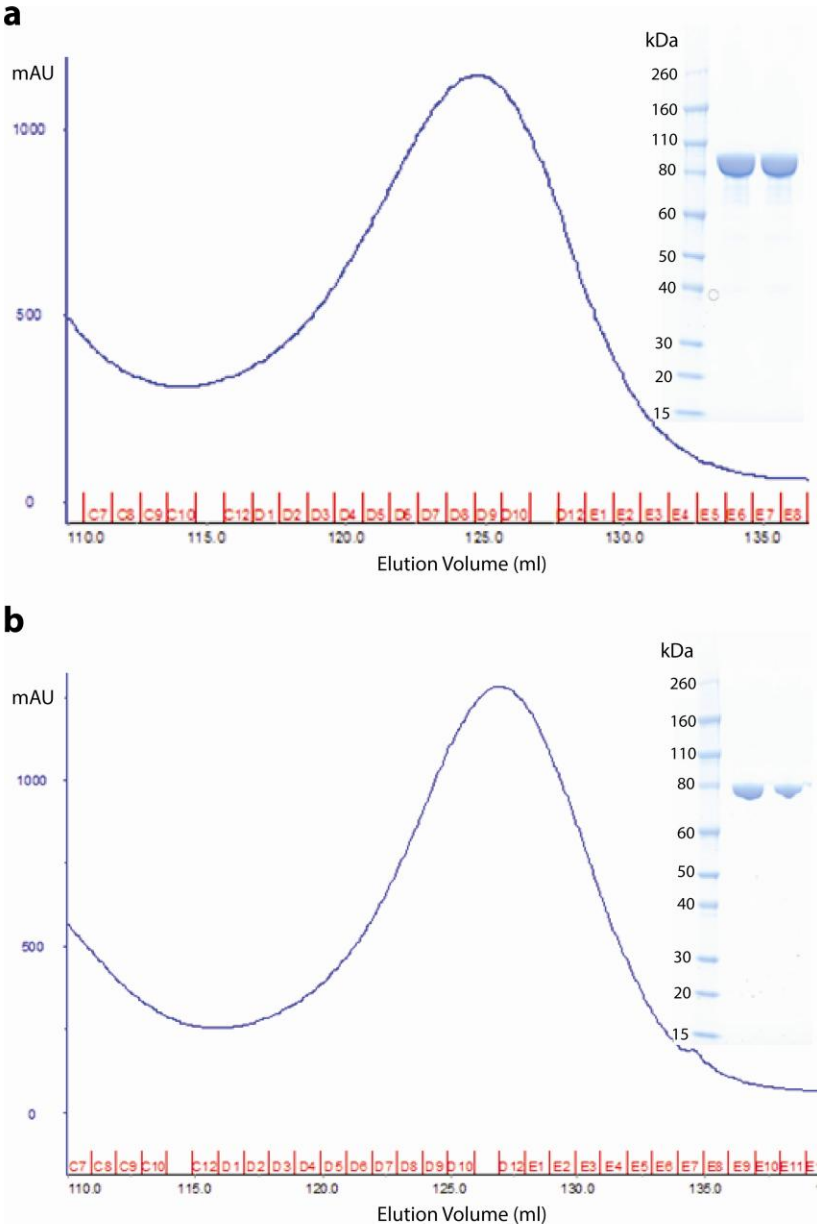

Supplementary Figure S1 Purification of MBP-SUFU-FL and MBP-SUFU-Δ for SAXS analysis. (*a*) Elution profile from size exclusion chromatography of MBP-SUFU-FL with SDS-PAGE analysis of fractions D9-D10 which were pooled and used for SAXS experiments. (*b*) Elution profile from size exclusion chromatography of MBP-SUFU-Δ with SDS-PAGE analysis of fractions D12-E1 which were pooled and used for SAXS experiments.

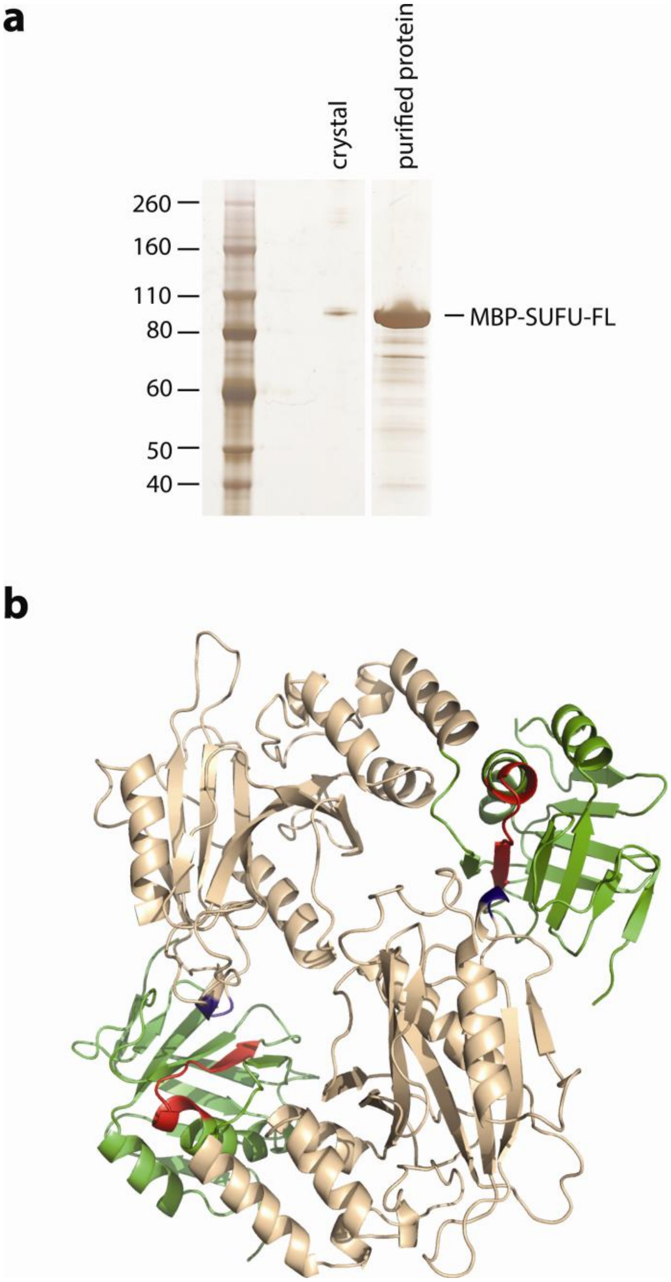

Supplementary Figure S2 Crystals of MBP-SUFU-FL. (*a*) Silver-stained SDS-PAGE analysis of crystallized MBP-SUFU-FL compared to purified protein shows the protein is intact. (*b*) Head-to-Tail arrangement of SUFU molecules in apo crystals. All apo crystal forms comprise pairs of SUFU molecules in which the N-terminal domain (beige) of one molecule packs against the C-terminal domain (green) of its partner. Residues WLG61-63 (purple) of SUFU make crystal contacts with residues in the proposed GLI-binding region (red) of the C-terminal domain.

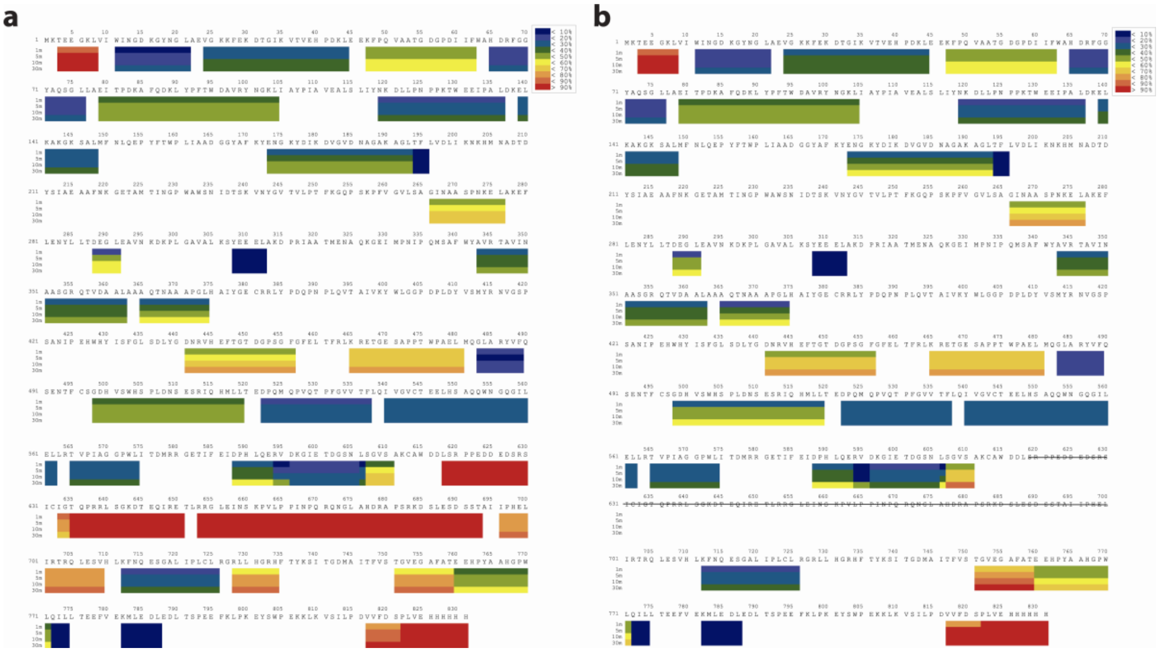

Supplementary Figure S3 Heat maps of MBP-SUFU-FL (*a*) and MBP-SUFU-Δ (*b*) at 1, 5, 10 and 30 min. Deuteration levels, color-coded according to the upper right text box, are shown below the corresponding amino acid sequence of MBP-SUFU-FL. Residue numbers of human SUFU can be obtained by subtracting 340 from those of MBP-SUFU-FL. Residues not present in the MBP-SUFU-Δ construct are indicated by a strikethrough. Because of the inherent characteristics of the HDX method, the first two amino acids at N-terminus of each peptic peptide are not taken into account when calculating deuterium incorporation.

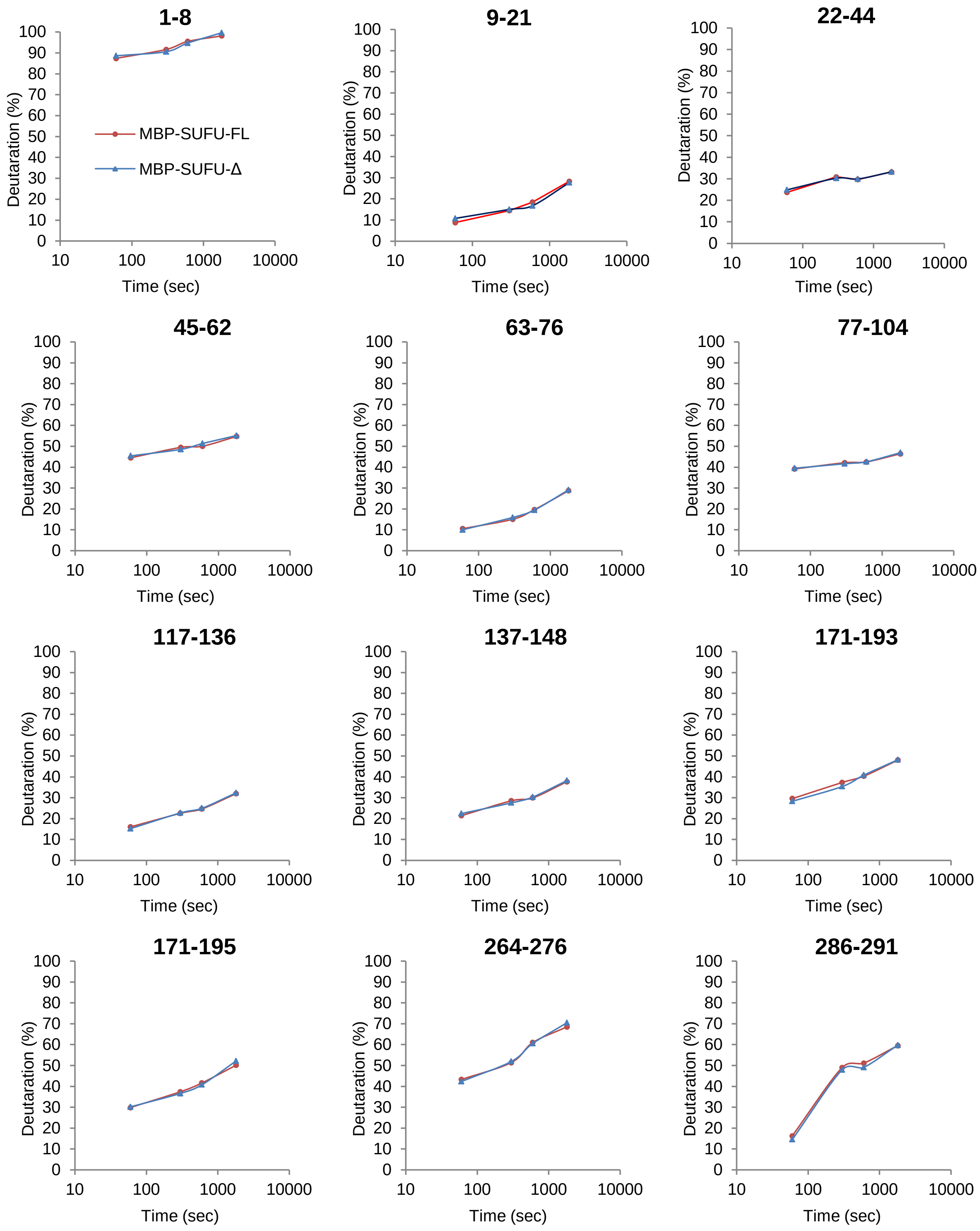
1-8
9-21
22-44
45-62
63-76
77-104
117-136
137-148
171-193
171-195
264-276
286-291
MBP-SUFU-FL
MBP-SUFU-Δ
Deutaration (%)
Time (sec)
0
10
20
30
40
50
60
70
80
90
100
10
100
1000
10000

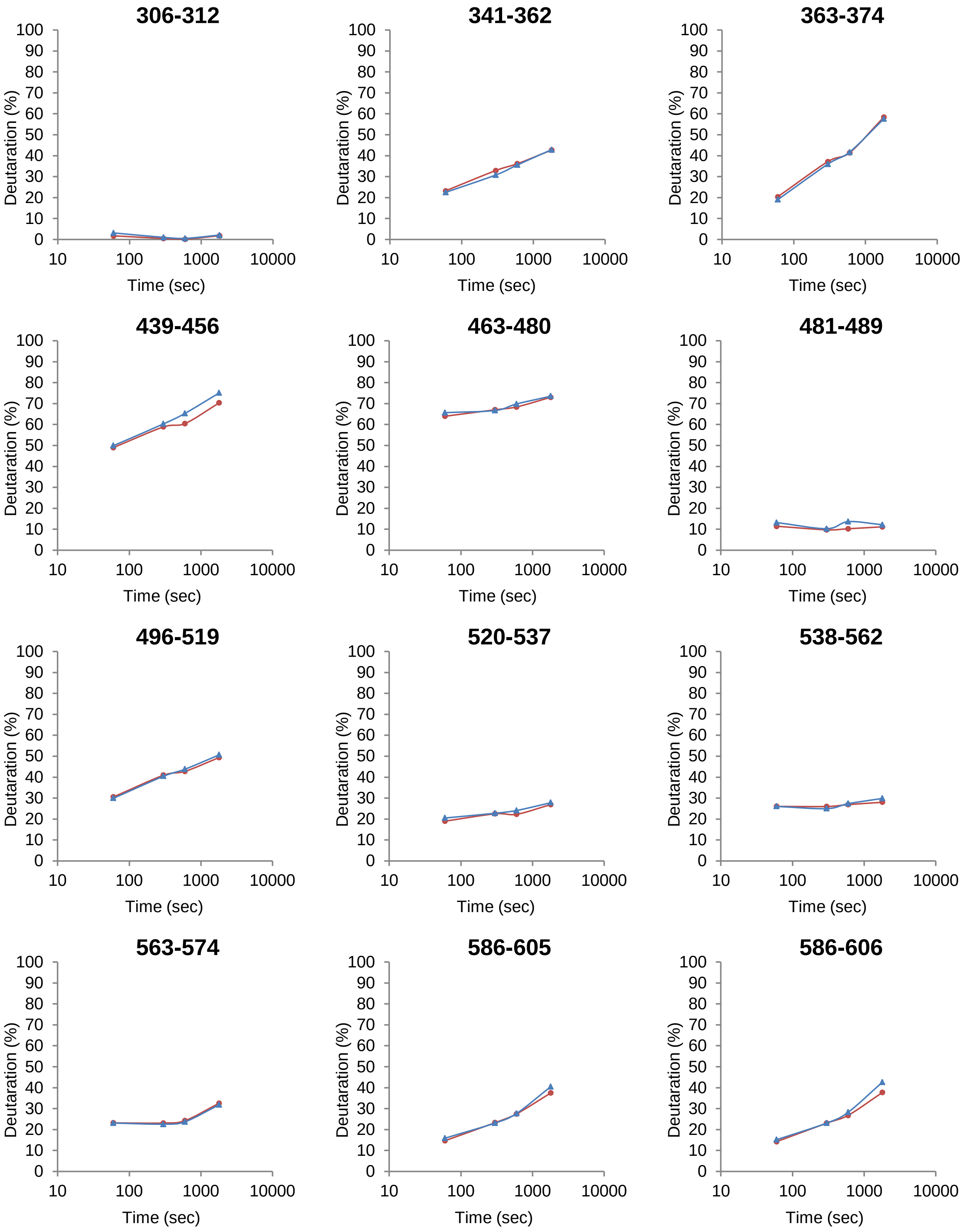
306-312
341-362
363-374
439-456
463-480
481-489
496-519
520-537
538-562
563-574
586-605
586-606
Deutaration (%)
Time (sec)
0
10
20
30
40
50
60
70
80
90
100
10
100
1000
10000

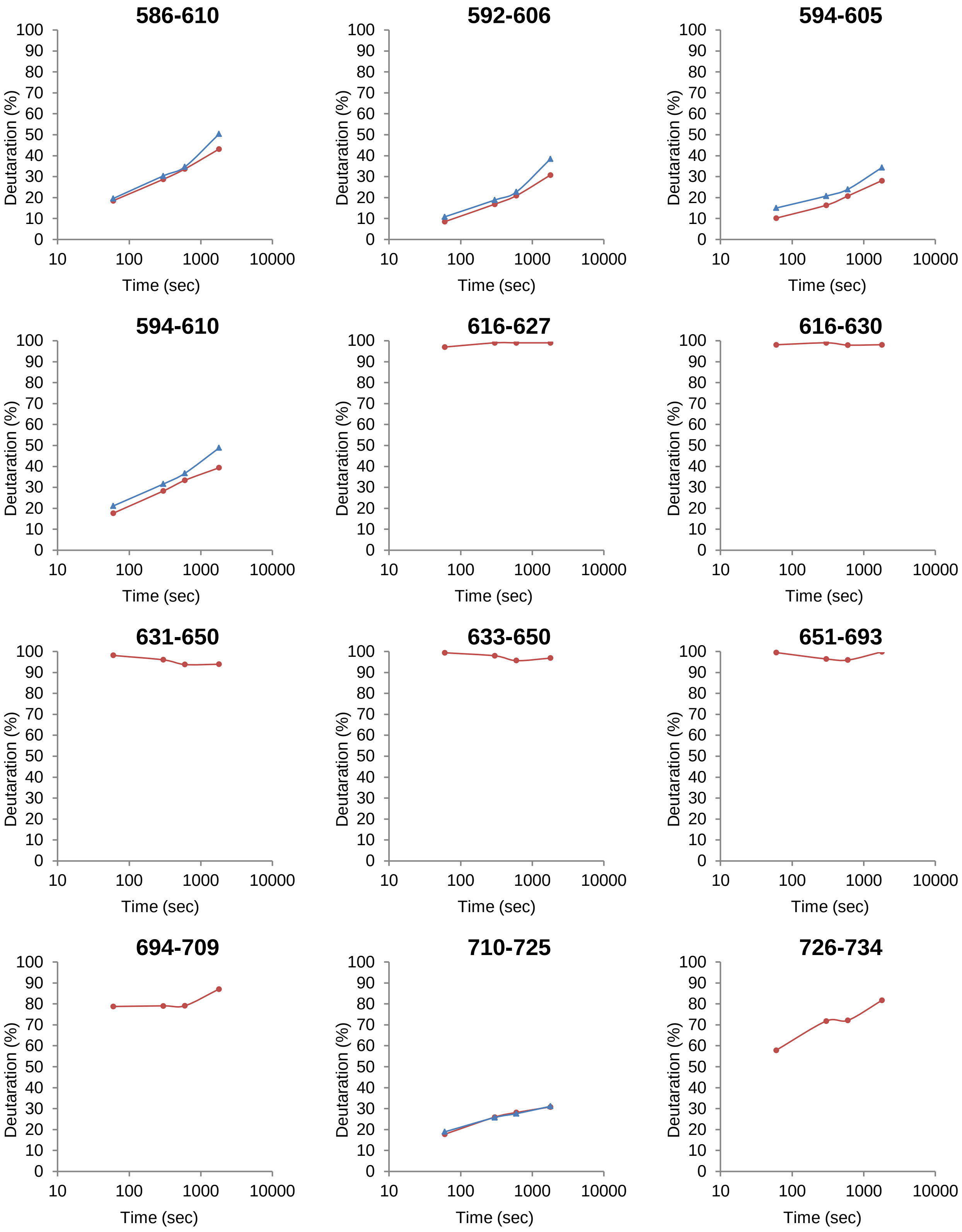
586-610
592-606
594-605
594-610
616-627
616-630
631-650
633-650
651-693
694-709
710-725
726-734
Deutaration (%)
0
10
20
30
40
50
60
70
80
90
100
Time (sec)
10
100
1000
10000

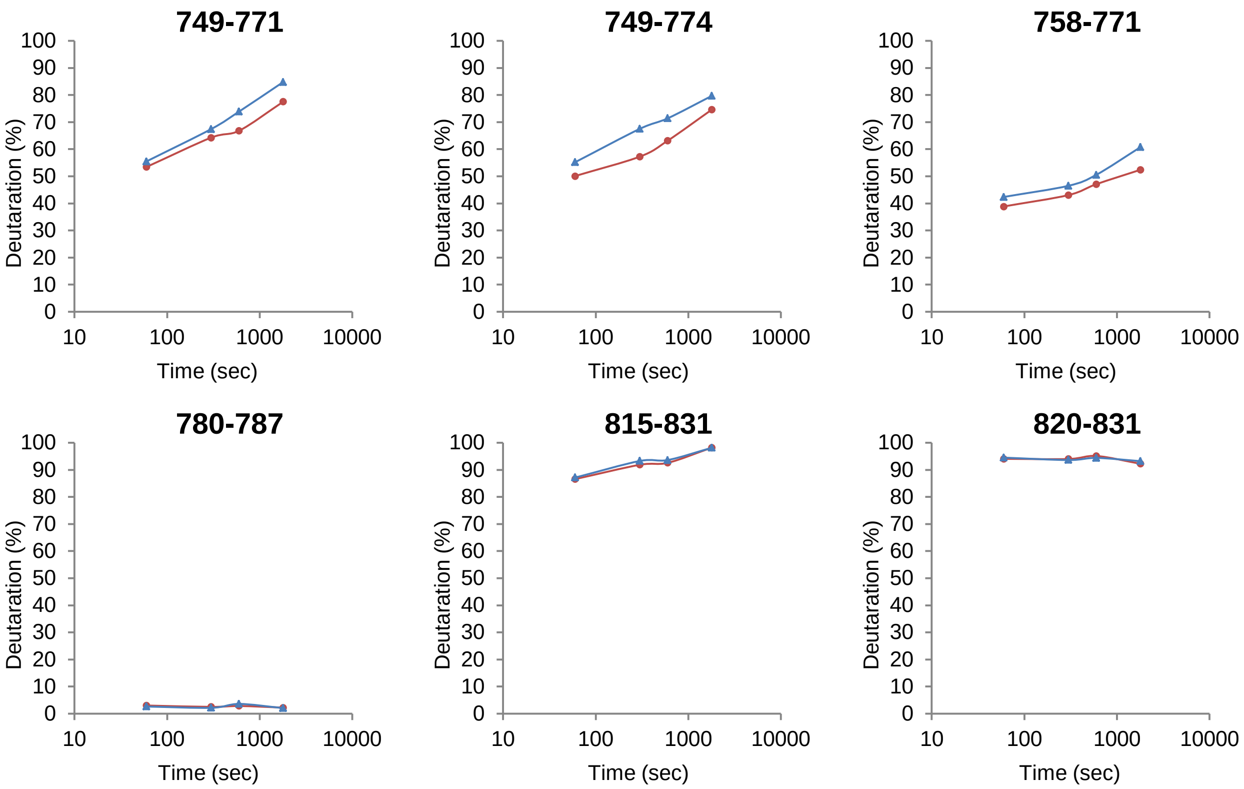

Supplementary Figure S4 Deuteration Plots of Individual Peptic Peptides Identified in MBP-SUFU-FL and MBP-SUFU-Δ Experiments. Red circles and blue triangles refer to MBP-SUFU-FL and MBP-SUFU-Δ, respectively. Residue numbering is as in Supplementary Figure 3. The kinetics of 42 high-confidence peptic peptides were calculated for MBP-SUFU-FL. 6 peptides corresponding to IDR residues 616-709 were fully deuterated at all time points; peptides corresponding to residues 594-610 and 749-774 show statistically significant lower deuteration in MBP-SUFU-FL than MBP-SUFU-Δ.

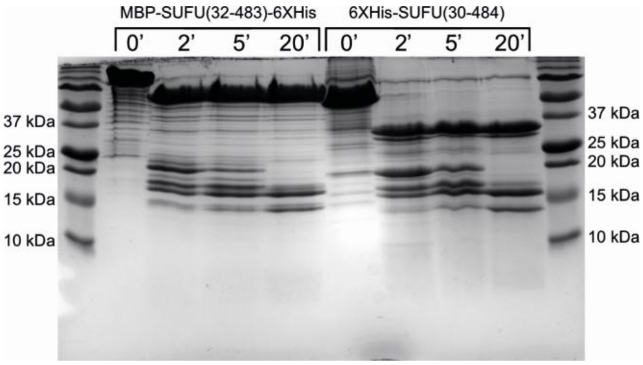

Supplementary Figure S5 Presence of an IDR is an inherent feature of human SUFU. A comparison of limited proteolysis patterns of *E. coli*-produced MBP-$SUFU_{32\text{-}483}$-6XHis and Sf9 cell-produced 6XHis-$SUFU_{30\text{-}484}$ over different lengths of time is shown.

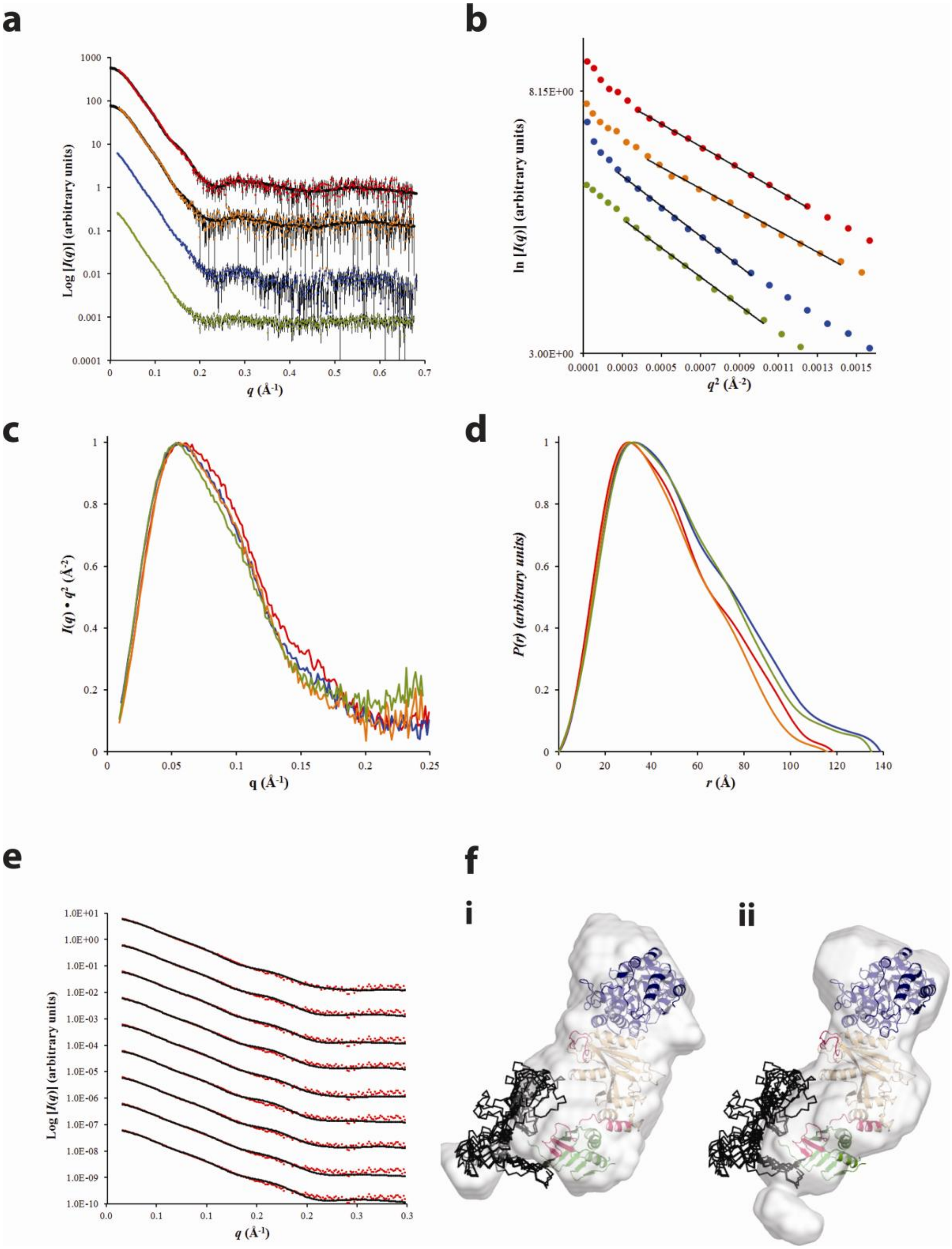

Supplementary Figure S6 SAXS analysis of MBP-SUFU-Δ and MBP-SUFU-FL. (*a*) SAXS profiles of 7.3 mg/ml MBP-SUFU-Δ (red), 4.5 mg/ml MBP-SUFU-Δ + $GLI1_p$ (orange), and 6.6 mg/ml MBP-SUFU-FL (blue) and 5.0 mg/ml MBP-SUFU-FL + $GLI1_p$ (green). The fit of the crystal structures of

apo and peptide-bound SUFU (10 and 8 models, respectively) to the SAXS data was calculated by CRYSOL. The fits with the lowest χ^2/χ^2_{free} (4.6/6.0 for apo and 1.7/2.3 for peptide-bound) are shown with black lines. (*b*) Linear portion of Guinier plots used to determine the *I*(0) and R_g values in Supplementary Table 2. In panels *a* and *b*, the data are scaled for easy visualization. (*c*) Normalized Kratky plots show clear peaks that return to baseline demonstrating that both proteins are folded. (*d*) Normalized *P(r)* plots calculated by GNOM and used for *ab initio* modeling. (*e*) Fits to the experimental SAXS data for 9 models of MBP-SUFU-FL generated by CORAL. (*f*) DAMMIF models of MBP-SUFU-FL cluster into two distinct groups, *i* and *ii*. CORAL-generated models of the IDR have some variability (as expected for an IDR), but an overall similarity to DAMMIF cluster 1.

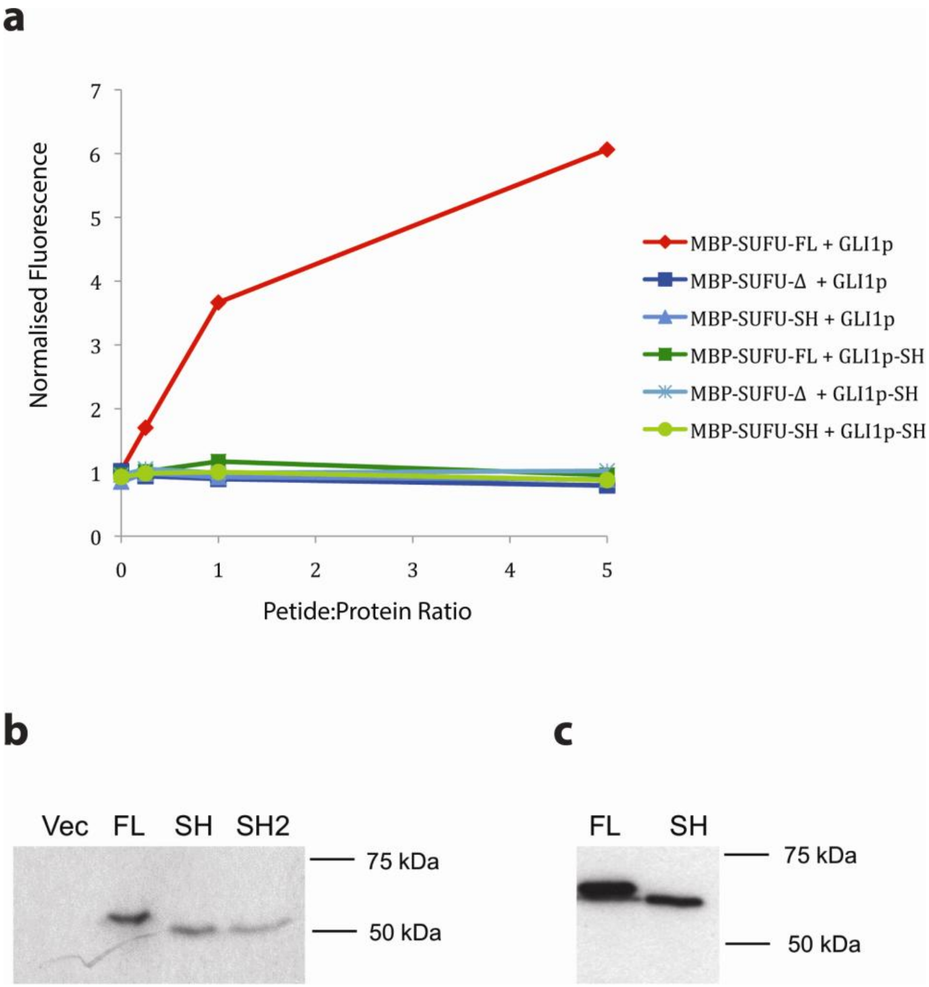

Supplementary Figure S7 SUFU IDR is characterized by unconventional structural properties. (*a*) $GLI1_p$ binding induces a change in the properties of SUFU-FL. Recombinant proteins MBP-SUFU-FL, MBP-SUFU-Δ and MBP-SUFU-SH were incubated with SYPRO Orange and the fluorescence measured with an excitation wavelength of 470 nm and an emission wavelength of 570 nm. $GLI1_p$ or $GLI1_p$-SH were added at the given peptide:protein ratios and fluorescence measured again. Measurements post-peptide addition were normalized by division with corresponding pre-peptide addition measurements. (*b*)-(*c*) Immunoblots showing different electrophoretic mobility between SUFU-FL (FL), SUFU-SH (SH) and SUFU-SH2 (SH2) expressed in mammalian cells *b*, as well as SUFU-FL and SUFU-SH expressed in bacteria *c.*

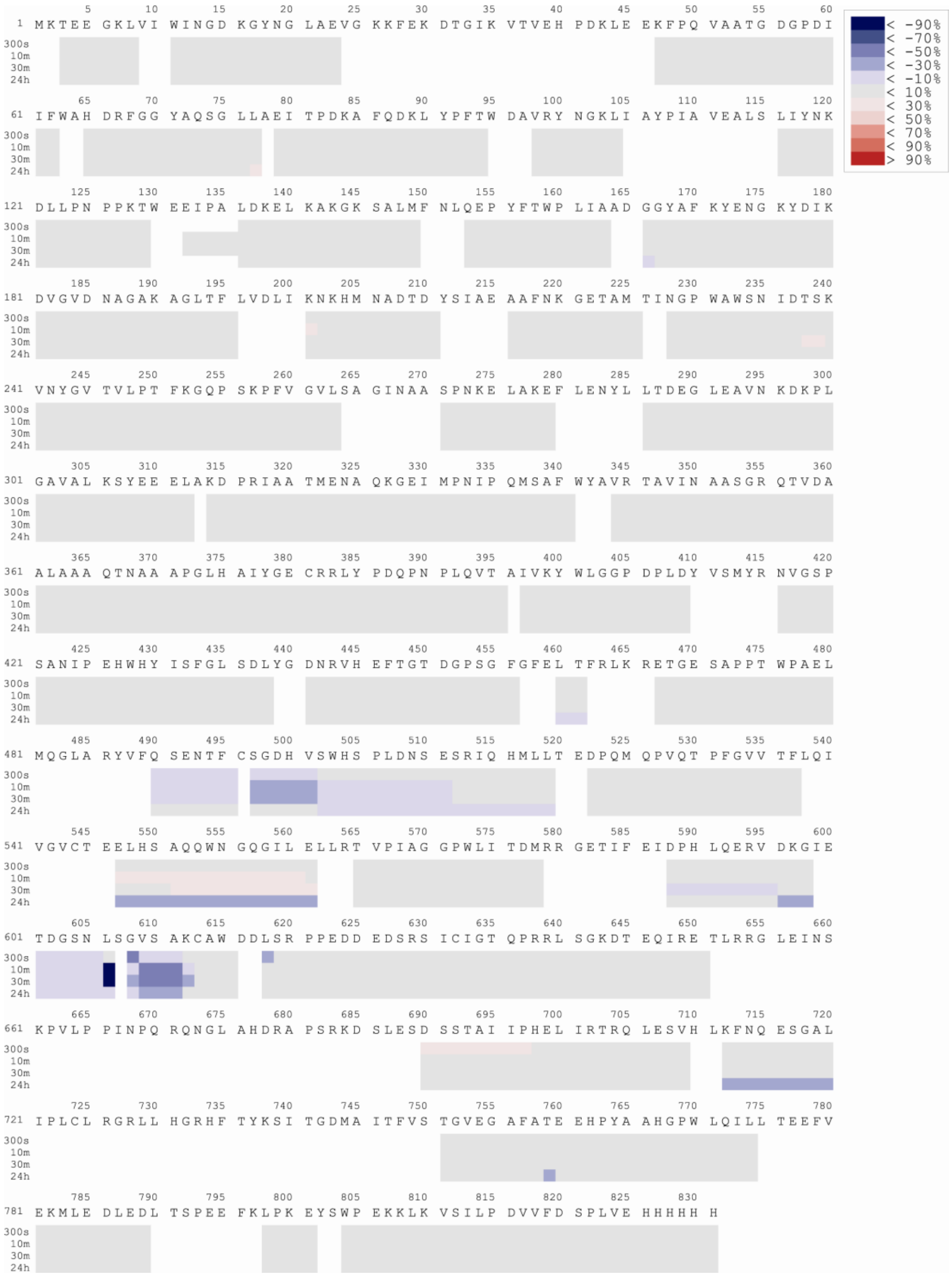

Supplementary Figure S8 HDX MS perturbation map of the MBP-SUFU-FL/GLI3$_p$ complex. Color-coded deuteration differences between MBP-SUFU-FL (free protein) and MBP-SUFU-FL/GLI3$_p$ complex are shown below the corresponding amino acid sequence at 300 s, 10 min, 30 min and 24 h.

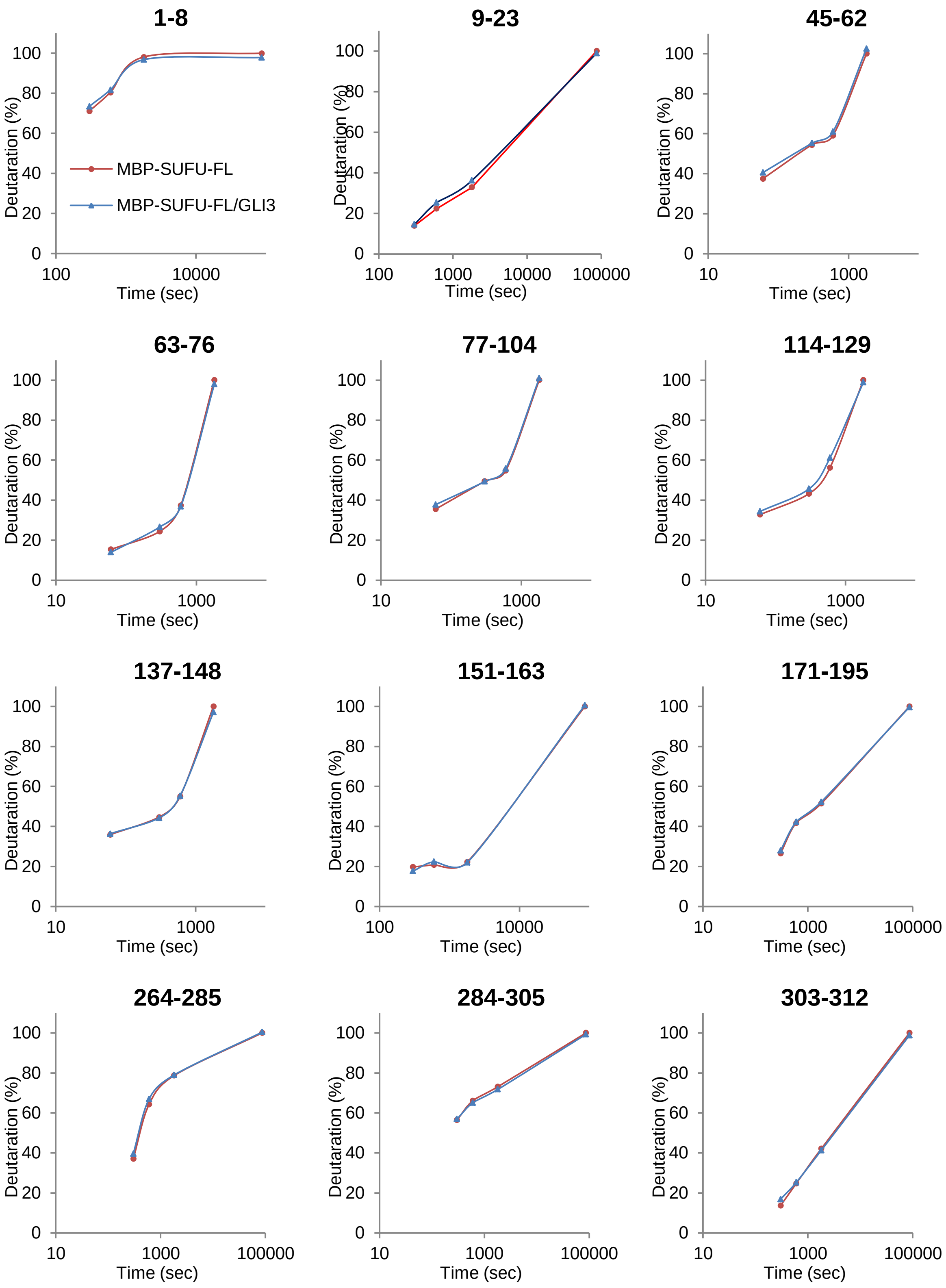
1-8
9-23
45-62
63-76
77-104
114-129
137-148
151-163
171-195
264-285
284-305
303-312
MBP-SUFU-FL
MBP-SUFU-FL/GLI3
Deutaration (%)
Time (sec)

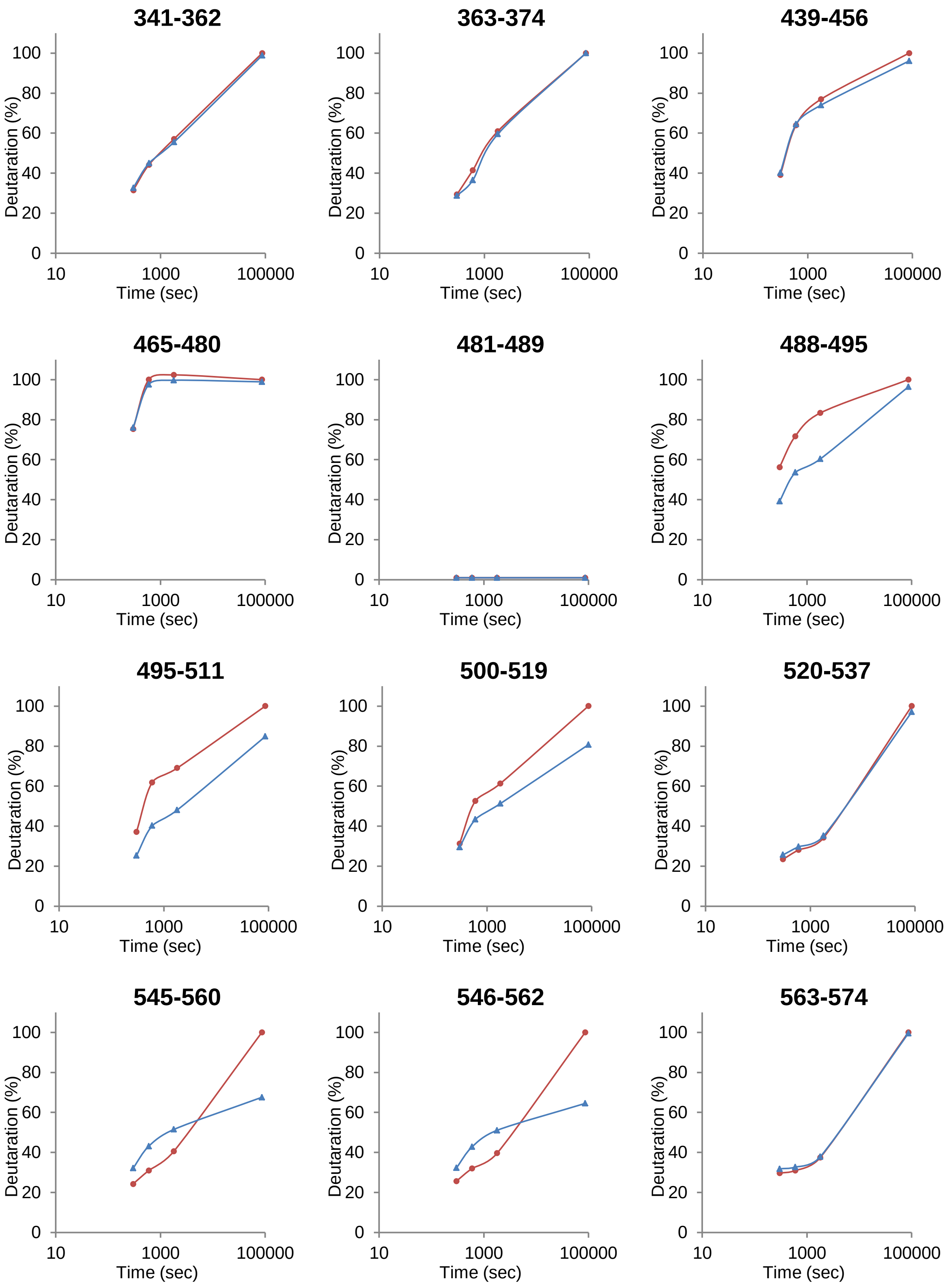
341-362
363-374
439-456
465-480
481-489
488-495
495-511
500-519
520-537
545-560
546-562
563-574
Deutaration (%)
0
20
40
60
80
100
Time (sec)
10
1000
100000

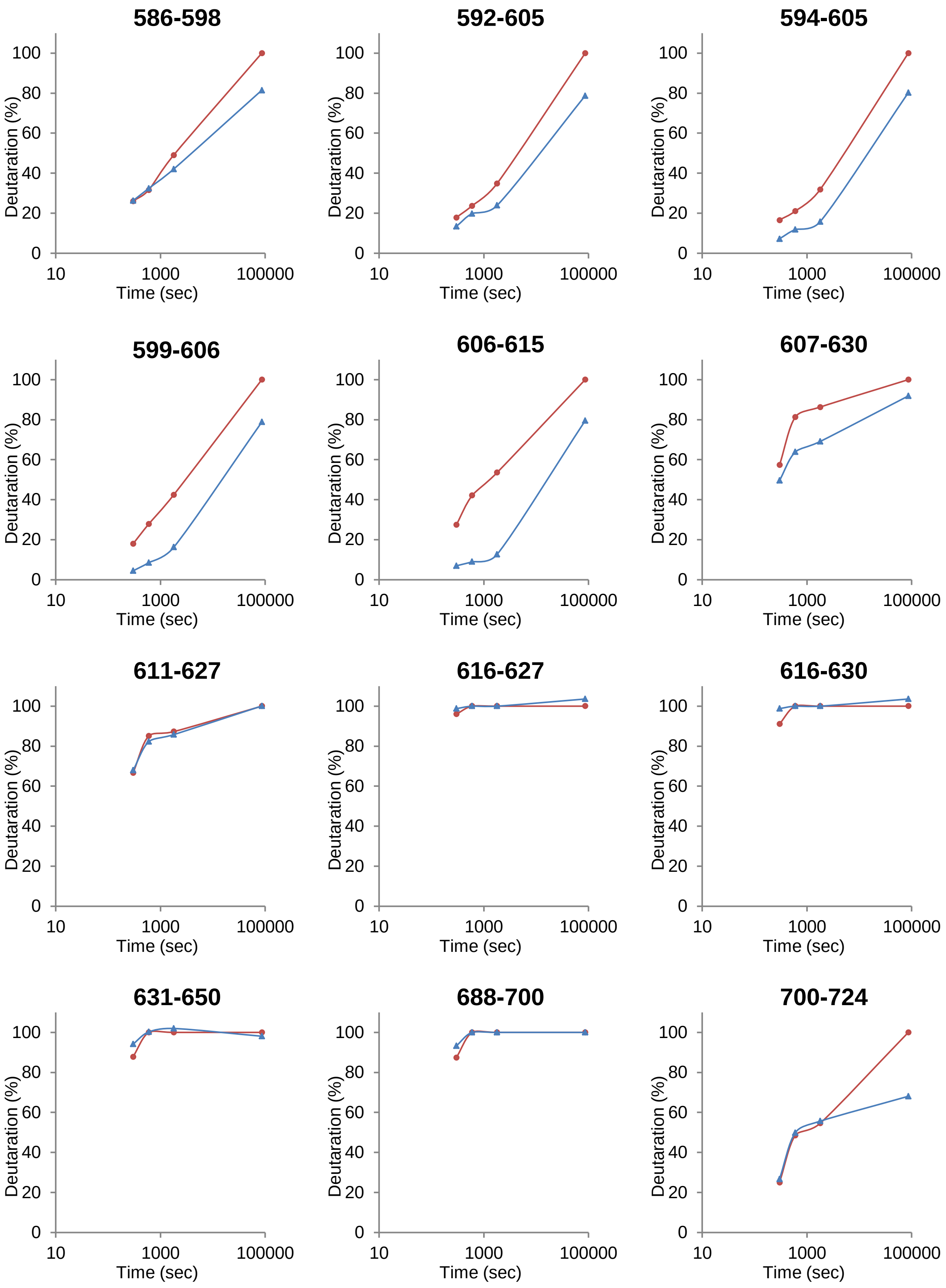
586-598
592-605
594-605
599-606
606-615
607-630
611-627
616-627
616-630
631-650
688-700
700-724
Deutaration (%)
0
20
40
60
80
100
10
1000
100000
Time (sec)

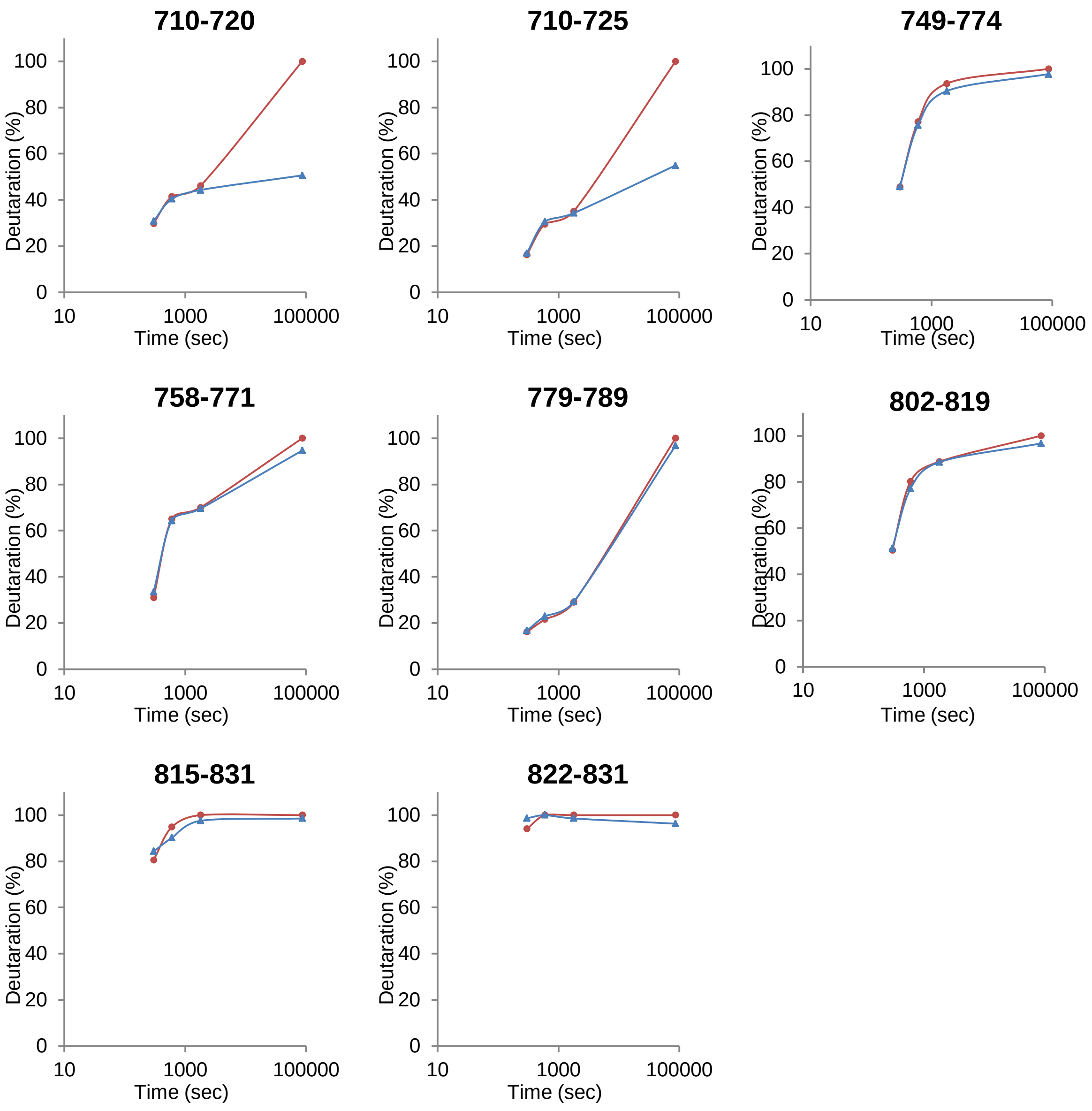

Supplementary Figure S9 Deuteration Plots of Individual Peptic Peptides Identified in MBP-SUFU-FL and MBP-SUFU-FL/GLI3$_p$ Experiments**.** Red circles and blue triangles refer to MBP-SUFU-FL and MBP-SUFU-FL/GLI3$_p$, respectively. Residue numbering is as in Figures S3 and S4. The kinetics of 44 high-confidence peptic peptides were calculated for MBP-SUFU-FL. Peptides corresponding to residues 488-511, 545-560, 592-611 and 700-720 show statistically significant lower deuteration in the MBP-SUFU-FL/GLI3$_p$ experiment than in the one lacking GLI3$_p$.

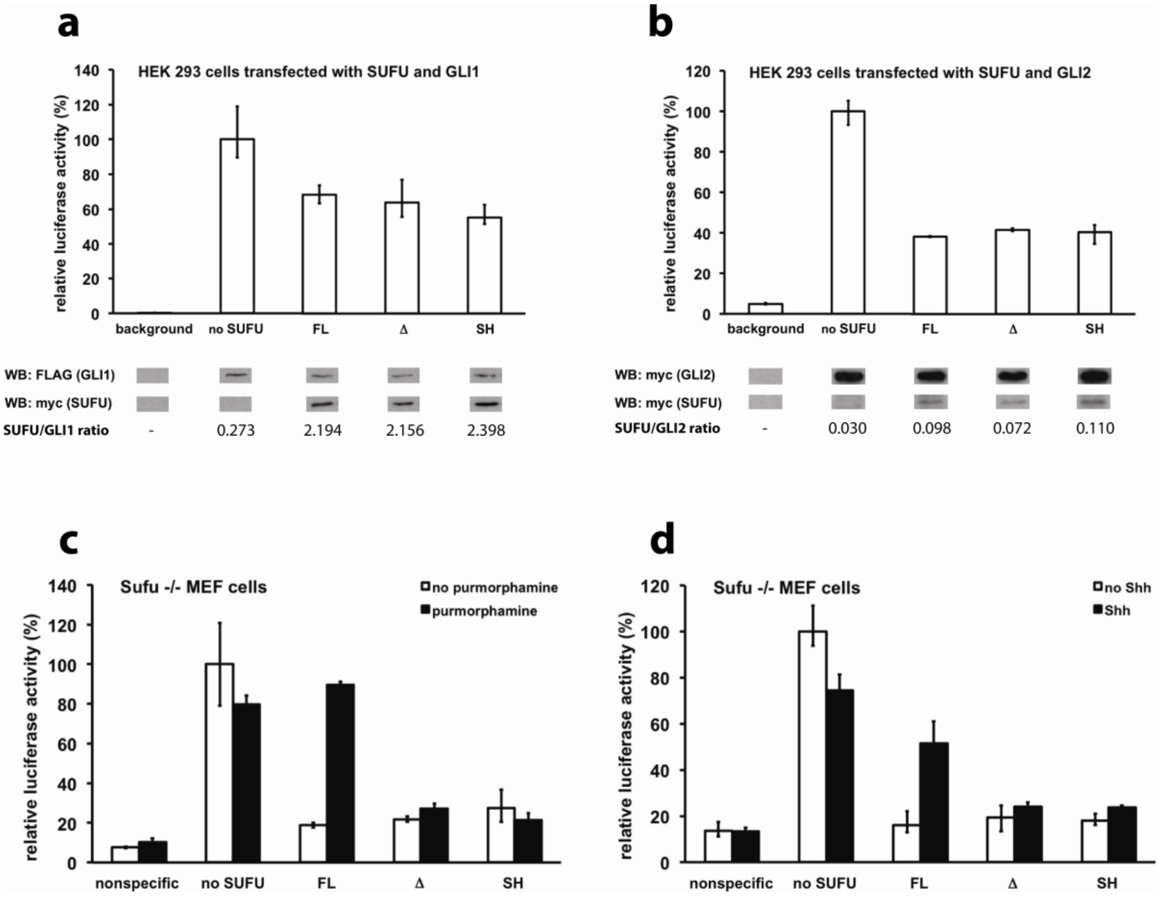

Supplementary Figure S10 The IDR regulates SUFU activity. (*a*)-(*b*), Reporter gene activity assays comparing the repression capacity of SUFU-FL (FL), SUFU-Δ (Δ) and SUFU-SH (SH) in HEK293 cells co-transfected with GLI1 or GLI2. Immunoblot analysis shows similar levels of

protein expression in transfected cells. Ratios of quantified band densities at the bottom confirm comparable SUFU/GLI ratios in the analyzed samples. Expression levels of SUFU variants were chosen to give partial repression of Hh pathway activity in order to sensitize the assay to detect potential differences between the SUFU variants. (*c*)-(*d*) Lack of Hh pathway reactivation by purmorphamine or Shh in *Sufu-/-* MEF cells expressing SUFU-Δ and SUFU-SH. Nonspecific denotes cells transfected with a mutant GLI reporter. Error bars indicate range of data in three parallel samples.

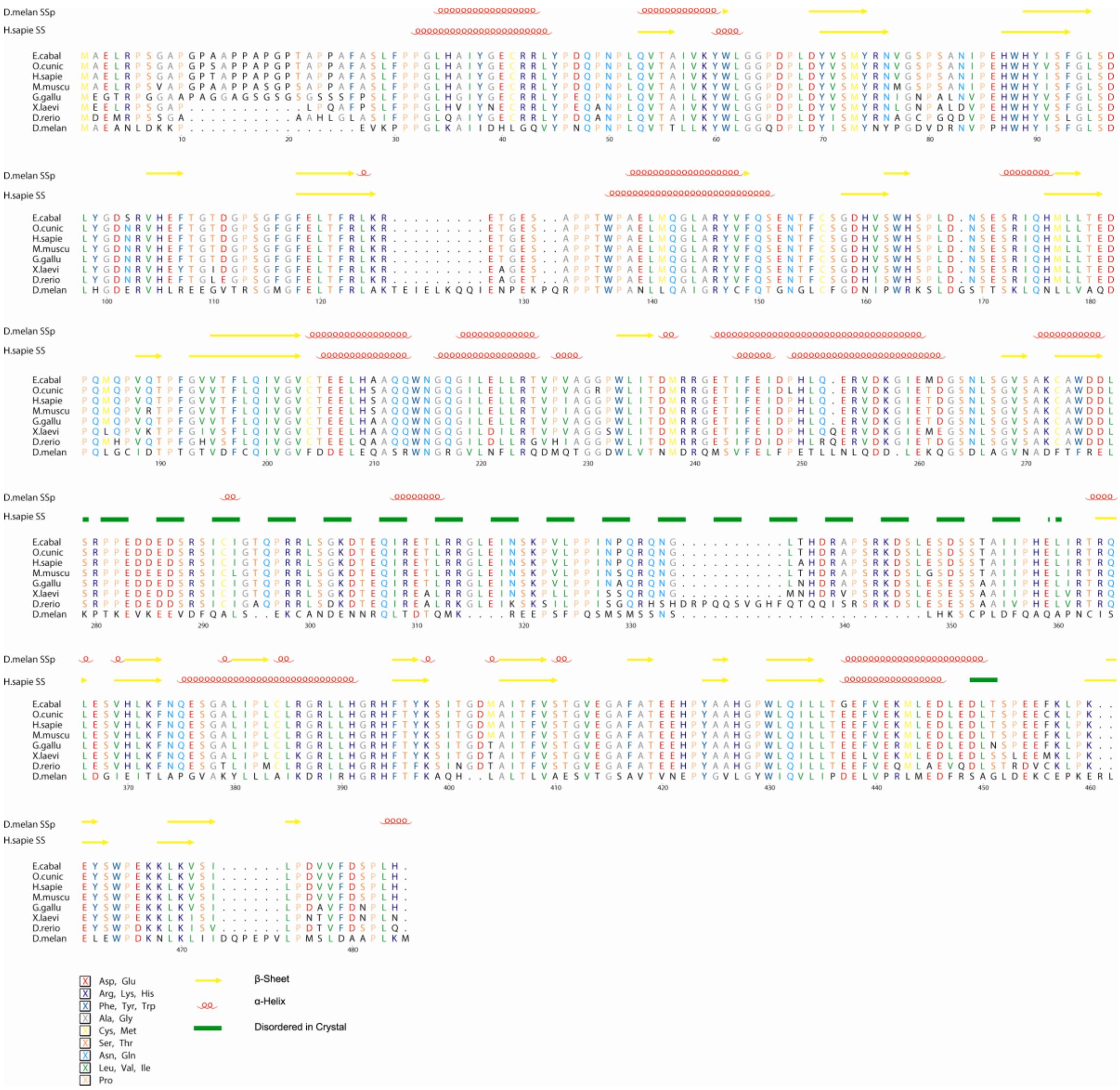

Supplementary Figure S11 Multiple sequence alignment of SUFU homologs from different species. Sequences were aligned using STRAP (Gille & Frömmel, 2001) and conserved residues colored according to the given scheme. Numbering is given for human SUFU. The secondary structure (SS) of human SUFU derived from the crystal structure of MBP-SUFU-FL, as well as the predicted secondary structure (SSp) of *Drosophila* sufu produced by SOPMA (Geourjon & Deléage, 1995), are shown.

Supplementary Table S1(a) SUFU IDR construct sequences

Construct sequences corresponding to residues 278-361 of human SUFU are shown.

FL: SUFU-FL; Δ: SUFU-Δ; SH: SUFU-SH; SH2: SUFU-SH2; IDR_{fly}, SUFU-IDR_{fly}.

FL	SRPPEDDEDSRSICIGTQPRRLSGKDTEQIRETLRRGLEINSKPVLPPINPQRQNGLAHDRAPSRKDSLESDSSTAIIPHEL
Δ	PSRGEDP
SH	LPSHRLTISQIEGESLIKLAISKSPISNRDGQPARSDNIHDPQLSLAERRPEPRLQCPTSDIKTNERPGDPERERDVRTDGS
SH2	RTISIPDRRQRTQLREQPDSHHQGLRSIPSEGSTNLDLEPNNRCIDLLRIDGSKAPIKVLTEDDESPPEIGRRKSPESAAPS
IDR_{fly}	KPTKEVKEEVDFQALSEKCANDENNRQLTDTQMKREEPSFPQSMSMSSNSLHKSCPLDFQAQAPNC

Supplementary Table S1(b) GLI peptide sequences

$GLI1_p$*	115 – TSPGGSYGHLSIGTMSP - 131
$GLI1_p$-SH*	115 – SGLYTGSGPSIPMGTSH - 131
FAM-$GLI1_p$#	115 - 5-FAM–TSPGGSYGHLSIGTMSP - 131
$GLI2_p$*	267 – SAASGSYGHLSAGALSP - 283
$GLI3_p$*	328 – SSASGSYGHLSASAISP - 344
$GLI3_p$-SHC#	328 – SSASGSYGH - 336
*Synthesized by Dr W. Mawby, University of Bristol<br>#Pepceuticals limited	

Supplementary Table S2 SAXS-derived structural parameters.

	MBP-SUFU-Δ			MBP-SUFU-Δ + Gli1			MBP-SUFU-FL			MBP-SUFU-FL + Gli1		
Concentration (mg ml^{-1})	7.3	3.65	1.83	4.5	2.3	1.1	6.6	3.3	1.65	5.0	2.5	1.25
**Structural parameters**												
I(0) (a.u.) [from *P*(*r*)]‡	5.95 ± 0.01	2.98 ± 0.01	1.42 ± 0.01	3.91 ± 0.01	2.00 ± 0.01	1.03 ± 0.01	6.81 ± 0.02	3.49 ± 0.01	1.69 ± 0.01	5.71 ± 0.02	2.95 ± 0.01	1.48 ± 0.01
R_g (Å) [from *P*(*r*)]	36.4 ± 0.1	36.7 ± 0.1	36.9 ± 0.2	35.0 ± 0.1	35.0 ± 0.1	35.5 ± 0.2	41.1 ± 0.1	41.3 ± 0.2	41.8 ± 0.3	39.9 ± 0.2	40.2 ± 0.2	40.5 ± 0.2
I(0) (a.u.) [from Guinier]	5.90 ± 0.02	2.98 ± 0.01	1.43 ± 0.01	3.96 ± 0.02	2.03 ± 0.01	1.06 ± 0.01	6.97 ± 0.03	3.62 ± 0.02	1.73 ± 0.01	5.89 ± 0.03	2.98 ± 0.02	1.50 ± 0.02
R_g (Å) [from Guinier]	35.6 ± 0.2	36.1 ± 0.2	36.7 ± 0.4	35.2 ± 0.2	35.4 ± 0.1	36.4 ± 0.4	41.1 ± 0.3	41.9 ± 0.3	41.8 ± 0.4	40.5 ± 0.2	39.6 ± 0.2	40.0 ± 0.6
D_{max} (Å)	119	118	119	116	115	116	139	140	140	135	135	135
Porod volume estimate (Å^3)	136038	134303	136365	147622	141309	135358	171035	171557	167566	185475	167346	171606
V_c (Å^2)†	590	596	621	600	580	589	718	724	752	710	693	682
Q_R (Å^3)†	9778	9840	10508	10227	9503	9531	12543	12510	13528	12447	12128	11628
**Molecular weight (M_r) determination**												
M_r (from Porod volume; Da)	85024	83939	85228	92264	88318	84599	106897	107223	104729	115922	104591	107254
M_r (from sequence; Da)	83961	83961	83961	85650	85650	85650	92385	92385	92385	94074	94074	94074
M_r (from Q_R; Da)†	79432	79935	85361	83079	77197	77425	101893	101625	109894	101112	98522	94459
**Software used**												
Primary data reduction	Rigaku SAXSLab											
Data processing	PRIMUS											
Ab initio analysis	DAMMIF											
Validation and averaging	DAMAVER											
Rigid-body modeling	CORAL											
Computation of model intensities	CRYSOL											
Graphical representations	PyMOL											

‡Intensities are not on an absolute scale and are reported in arbitrary units.

†Calculated according to Rambo & Tainer (2013) using the program SCATTER (https://bl1231.als.lbl.gov/scatter/)

Supplementary Table S3 Hydrogen bonding interactions between GLI1$_p$ and GLI3$_p$ side chains and MBP$_{A216H_K220H}$-SUFU-Δ$_{W61D_L62S_G63F_P453A_Δ454-456_K457A}$

GLI3 Residue	SUFU Residue	Distance (Å)			
		Chain A	Chain B	Chain C	Chain D
**H336**	**Y147**	3.18	2.67	-	3.26
**H336**	**D159**	3.11	-	3.19	-
**S338**	**E376**	2.73	3.15	3.00	3.10

SUFU	GLI1	Distance (Å)				GLI3	Distance (Å)			
		Chain					Chain			
		A	B	C	D		A	B	C	D
**Y147**	**H123**	2.85	2.59	3.09	2.97	**H336**	3.18	2.67	-	3.26
**D159**	**H123**	3.11	-	3.13	-	**H336**	3.11	-	3.19	-
**E376**	**S125**	2.74	-	3.14	3.01	**S338**	2.73	3.15	3.00	3.10

References

Geourjon, C. & Deléage, G. (1995). *Comput. Appl. Biosci.* **11**, 681–684.

Gille, C. & Frömmel, C. (2001). *Bioinformatics*, **17**, 377–378.
